# Phosphoantigen-driven dissociation of butyrophilin oligomers activates γδ T cells

**DOI:** 10.1038/s41421-026-00911-7

**Published:** 2026-08-11

**Authors:** Weizhi Xin, Bangdong Huang, Weijie Gao, Wenjia Zhang, Yundi Hu, Yuehua Liu, Enyuan Liang, Jin Chen, Yigong Shi, Qiang Su, Qiang Zhou

**Affiliations:** 1 Fudan University, Shanghai, China; 2 Research Center for Industries of the Future, Center for Infectious Disease Research, Zhejiang Key Laboratory of Structural Biology, State Key Laboratory of Gene Expression, School of Life Sciences, Westlake University, Hangzhou, Zhejiang China; 3 Westlake Laboratory of Life Sciences and Biomedicine, Hangzhou, Zhejiang China; 4 Institute of Biology, Westlake Institute for Advanced Study, Hangzhou, Zhejiang China; 5 Institute of Bio-Architecture and Bio-Interactions (IBABI), Shenzhen Medical Academy of Research and Translation (SMART), Shenzhen, Guangdong China; 6 Beijing Advanced Innovation Center for Structural Biology, Tsinghua-Peking Joint Center for Life Sciences, School of Life Sciences, School of Medicine, Tsinghua University, Beijing, China

**Keywords:** Immunology, Structural biology

## Abstract

γδ T cells represent a promising avenue for cancer immunotherapy. The Vγ9Vδ2 T-cell receptor (TCR), which is expressed by the predominant subset of γδ T cells, responds to phosphoantigen (pAg)-engaged butyrophilins (BTNs) on various cancer cells. However, the molecular mechanism underlying the pAg-mediated activation of Vγ9Vδ2 TCRs remains a subject of debate. Here, we employed an integrative approach to elucidate the mechanism of pAg reactivity in Vγ9Vδ2 T cells. Our results demonstrate that BTNs form higher-order oligomers in the absence of pAg. Upon pAg binding, these higher-order oligomers dissociate into separate tetramers, enabling Vγ9Vδ2 TCR engagement. This pAg-induced dissociation of higher-order BTN oligomers is critical for pAg-mediated activation of γδ T cells. Our findings reveal a mechanism of BTN higher-order oligomer dissociation-driven pAg sensing, providing valuable insight for future immunotherapeutic strategies.

## Introduction

γδ T cells are a unique population of immune cells that can recognize and kill tumors^1^. Unlike αβ T cells, which recognize neoantigenic peptides presented by major histocompatibility complex (MHC) molecules on tumor cells, γδ T cells can detect a wide range of ligands in an MHC-independent manner^2–4^. This characteristic is particularly advantageous in certain malignancies in which MHC expression is downregulated and the mutational burden is low, such as pancreatic adenocarcinoma^5^. As a result, γδ T cells have emerged as promising candidates for cancer immunotherapy, especially for tumors with reduced MHC expression or limited neoantigen presentation^5^.

In humans, Vγ9Vδ2 T cells constitute the predominant subset of circulating γδ T cells, accounting for 1%–5% of total blood T cells^6^. During certain bacterial and parasitic infections, the population of Vγ9Vδ2 T cells can expand dramatically, reaching up to 50% of circulating T cells^7,8^. Vγ9Vδ2 T cells exhibit a range of effector functions, including lysing tumor cells, producing cytokines and chemokines, and priming αβ T cells via antigen presentation^2,3^. Owing to their distinct roles in tumor immunity, Vγ9Vδ2 T cells are promising targets for cancer immunotherapy^5^.

Vγ9Vδ2 T cells are activated by small non-peptidic phosphorylated metabolites referred to as phosphoantigens (pAgs)^9,10^. pAgs are naturally produced by microorganisms through the non-mevalonate pathway, such as (E)-4-hydroxy-3-methyl-but-2-enyl-pyrophosphate (HMBPP)^11,12^, or by human cells via the mevalonate pathway, as in the case of isopentenyl pyrophosphate (IPP)^10,12^. In the tumor microenvironment, dysregulation of the mevalonate pathway leads to IPP accumulation, which triggers Vγ9Vδ2 T-cell activation^13–15^. Aminobisphosphonates, such as zoledronate, enhance the intracellular accumulation of endogenous pAgs and are used in certain clinical trials to boost γδ T-cell activity in cancer patients^16,17^.

The activation of Vγ9Vδ2 T cells by pAgs depends on BTN3A1 and BTN2A1 molecules^18–20^. Notably, recent studies have demonstrated the essential roles of BTN3A2 or BTN3A3 in pAg responses^21–25^. BTN3A2 or BTN3A3 is required for the cell surface expression of BTN2A1 and BTN3A1. Genetic knockout of both *BTN3A2* and *BTN3A3* abolishes Vγ9Vδ2 T-cell activation, indicating that BTN3A2 or BTN3A3 is indispensable in the recognition of the BTN complex by the Vγ9Vδ2 T-cell receptor (TCR).

BTN2A1, BTN3A1, and BTN3A3 exhibit similar structural features, comprising an IgG-like variable (IgV) domain and an IgG-like constant (IgC) domain in the extracellular region, a transmembrane (TM) domain, a juxtamembrane (JM) domain and a B30.2 domain in the intracellular region^26,27^. In contrast, BTN3A2 lacks the intracellular B30.2 domain. Previous studies have elucidated the interactions between BTN molecules and the Vγ9Vδ2 TCR, demonstrating that the IgV domain of BTN2A1 binds to the Vγ9 domain of the TCRγ chain^19,20^. Furthermore, the BTN2A1 homodimer forms a tetrameric complex with the BTN3A1/3A2 or BTN3A1/3A3 heterodimer^28,29^. Recent studies have proposed two models for pAg-mediated T-cell activation^28,29^. The first model describes a “plier-like gripping” mechanism in which pAg facilitates the opening of the BTN ectodomain, enabling Vγ9Vδ2 TCR binding and subsequent T-cell activation^29^. The second model suggests that pAg promotes the dimerization of BTN2A1 and BTN3A1 to form a tetrameric complex that engages the Vγ9Vδ2 TCR and initiates activation^28^.

Currently, the molecular mechanisms underlying Vγ9Vδ2 T-cell activation by pAgs remain a subject of debate. In this study, we used a mammalian co-expression system to reconstitute full-length, membrane-associated BTN complexes containing BTN2A1, BTN3A1, and either BTN3A2 or BTN3A3. This approach preserves the native architecture of BTN assemblies and provides a more physiologically relevant framework for assessing their interactions with the Vγ9Vδ2 TCR than studies using their extracellular domains. With this system, we determined a series of cryo-electron microscopy (cryo-EM) structures of full-length BTN molecules. Through biochemical, biophysical, and cellular analyses, we demonstrated that BTN molecules assemble into higher-order oligomers on the cell membrane. Notably, pAg binding triggers the dissociation of these oligomers into tetramers, facilitating Vγ9Vδ2 TCR binding and the subsequent activation of Vγ9Vδ2 T cells.

## Results

### BTN molecules form higher-order oligomers in the absence of pAgs

To explore the molecular organization of BTN molecules, we fused affinity tags to the C-termini of BTN2A1 and BTN3A1 and co-expressed them with BTN3A2 or BTN3A3 in mammalian cells (Supplementary Fig. S1a). After tandem affinity purification, we subjected the purified complexes to size exclusion chromatography in the absence of pAgs (Supplementary Fig. S1b). For clarity, we designated the BTN2A1/3A1/3A2 complex as the 3A2 complex and the BTN2A1/3A1/3A3 complex as the 3A3 complex.

**Fig. 1:**
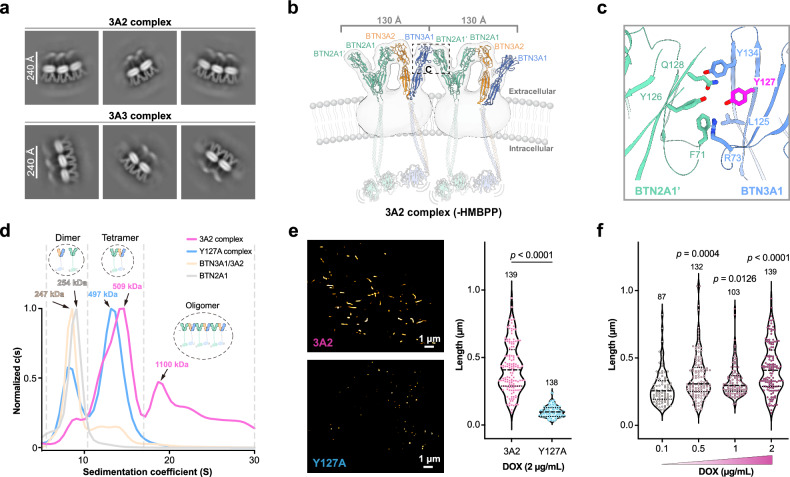
BTN molecules form higher-order oligomers on the cell membrane. **a** 2D class averages demonstrating that both the WT 3A2 complex (top panel) and the WT 3A3 complex (bottom panel) form oligomers in the absence of HMBPP. **b** Docking of two ectodomains of the 3A2 complex into the cryo-EM maps of the WT 3A2 complex oligomer. The cryo-EM map is contoured at 3 σ. The ectodomains of the 3A2 complex were flexibly docked into the map using the Namdinator server (https://namdinator.au.dk). Flexible transmembrane and cytoplasmic regions (unresolved cryo-EM density) are shown as transparent cartoons. **c** Y127^BTN3A1^ is located in the IgV^BTN2A1^–IgV^BTN3A1^ interface (PDB ID: 8DFX^33^). **d** AUC reveals distinct sedimentation profiles of the 3A2 complex, BTN2A1, and Y127A complex in the absence of HMBPP. Peaks at ~497–508 kDa correspond to the tetrameric assembly embedded in micelles, whereas the peak at ~1100 kDa corresponds to higher-order oligomers. Y127A, 3A2 complex variant harboring a Y127A mutation in BTN3A1. The raw data were normalized to 0–1 in GraphPad. **e** Left panel: zoomed TIRF-SIM images of cells expressing the WT 3A2 complex or Y127A complex. Right panel: violin plots of the length distribution for filaments identified in the SIM reconstruction. The WT 3A2 complex in cells induced by 2 µg/mL DOX assembles into filaments containing ~7–85 repeats of the tetrameric unit. *P* values were determined by the Mann‒Whitney test. DOX, doxycycline. **f** Violin plots of the length distribution of filaments identified in the SIM reconstruction of cells expressing the WT 3A2 complex induced with different concentrations of DOX. *P* values were determined by the Kruskal‒Wallis test with Dunn’s multiple comparisons post-test. Lines indicate median values. The data corresponding to the 3A2 complex treated with 2 µg/mL DOX in panel (**e**) are identical to those of the 3A2 complex treated with 2 µg/mL DOX shown in panel (**f**).

We analyzed the structures of the 3A2 and 3A3 complexes in the absence of pAgs using cryo-EM (Supplementary Fig. S1c, d). Both complexes can form higher-order oligomers without HMBPP that consist of multiple tetrameric 3A2 or 3A3 complexes arranged side-by-side, with tightly packed ectodomains (Fig. 1a). The intracellular regions appeared obscure, indicating substantial conformational flexibility in the JM and B30.2 domains in the absence of HMBPP (Fig. 1a).

Using our standard data processing pipeline^30–32^, we obtained a cryo-EM map of moderate resolution (Supplementary Fig. S1e–g). As shown in the two-dimensional (2D) averages, the intracellular regions were unresolved, suggesting substantial conformational flexibility in the JM and B30.2 domains of the oligomer (Fig. 1b). Flexible docking of the ectodomain structures of the tetrameric 3A2 complex (described later) into the map revealed a side-by-side arrangement of the two 3A2 complexes, likely bridged by the IgV domains of BTN3A1 and BTN2A1’, which is consistent with the recently reported crystal structure^33^ (Fig. 1b). Extensive van der Waals interactions are observed at the interface between IgV_BTN2A1’_ and IgV_BTN3A1_. Notably, Tyr127 plays a critical role in this interface, as the Y127A mutation abolished the interaction between BTN2A1 and BTN3A1 ectodomains^33^ (Fig. 1c).

To investigate whether BTN molecules assemble into higher-order oligomers in the absence of pAgs, we assessed their oligomerization state in solution by analytical ultracentrifugation (AUC). Purified BTN2A1 and BTN3A1/BTN3A2 displayed peaks corresponding to molecular weights of 254 kDa and 247 kDa, respectively (Fig. 1d), which are consistent with the homodimeric and heterodimeric configurations. The purified 3A2 complex exhibited a peak at 509 kDa, which matched the expected tetrameric assembly. However, additional higher molecular weight species (HMWS), including a prominent peak at ~1100 kDa (Fig. 1d), were also detected, suggesting the presence of oligomers larger than the tetrameric form. Notably, the Y127A mutation in BTN3A1 (referred to as Y127A), which was predicted to interfere with higher-order oligomerization (Supplementary Fig. S1b, c), selectively eliminated the HMWS while maintaining the tetrameric peak (Fig. 1d). These findings provided biochemical evidence supporting the higher-order oligomerization of BTN without pAgs.

To further confirm the formation of higher-order BTN oligomers on the cell membrane, we performed super-resolution imaging using total internal reflection fluorescence structured illumination microscopy (TIRF-SIM) in NIH-3T3 cells expressing either wild-type (WT) or mutant 3A2 complex variants (Fig. 1e, left). TIRF-SIM imaging revealed that the WT 3A2 complex forms linear, filamentous clusters on the cell membrane, with lengths ranging from ~100 nm to 1000 nm. In contrast, the Y127A complex formed small, homogeneous puncta with significantly shorter clusters, in which the median length was less than 100 nm (Fig. 1e, right). Given that each tetrameric unit spans ~130 Å (Fig. 1b), the observed clusters correspond to ordered arrays of 8–80 repeating tetrameric 3A2 complex units. These results demonstrate that the overexpressed 3A2 complex assembles into extended, hundreds of nanometer-scale filaments on the cell membrane and that the IgV_BTN3A1_–IgV_BTN2A1’_ interface is critical for this oligomerization.

To determine whether 3A2 complex oligomerization is concentration dependent, we generated cell lines with inducible and tunable expression of the 3A2 complex. Reducing the concentration of doxycycline led to a modest decrease in the median length of the filamentous clusters (Fig. 1f). However, the predominant population of filamentous clusters remained above 250 nm in length, indicating that even at low expression levels, the 3A2 complex retains the capacity to form higher-order oligomers on the cell membrane. This analysis revealed that filament formation is relatively consistent across varying expression levels and appears to be an intrinsic property of BTN molecules on the cell membrane.

### PAgs drive the dissociation of higher-order BTN oligomers

**Fig. 2:**
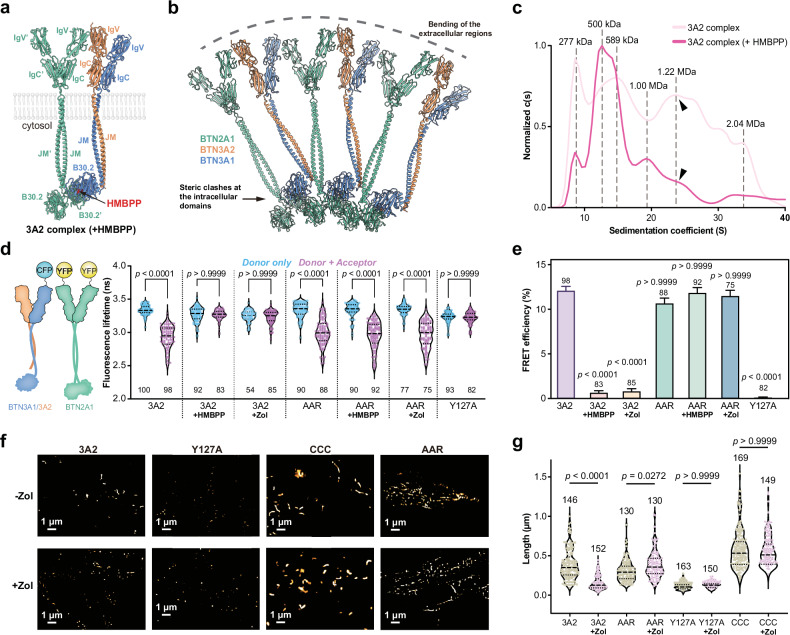
PAgs induce the dissociation of BTN higher-order oligomers. **a** The full-length structure of the HMBPP-bound 3A2 complex. The complex consists of a BTN2A1 homodimer and a BTN3A1/BTN3A2 heterodimer. BTN2A1 interacts with BTN3A2 via IgV domains on the extracellular side and with BTN3A1 via B30.2 domains on the intracellular side. The HMBPP is shown in sphere representation in red. **b** Multiple HMBPP-bound 3A2 complexes were concatenated on the basis of the IgV_BTN2A1_–IgV_BTN3A1_ interface (PDB ID: 8DFX^33^). Binding of HMBPP induces bending of the extracellular region of the BTN complex and causes spatial conflicts at the intracellular domains, which eventually destabilize higher-order oligomeric assembly. **c** AUC sedimentation profiles of the WT 3A2 complex ( ± 40 μM HMBPP). HMBPP drives the dissociation of high-mass oligomers into lower-mass species. The raw data were normalized to 0–1 in GraphPad with two aberrant points removed. **d** FLIM-FRET assays assessing the interaction between BTN2A1 and BTN3A1. Cartoon depicting the construct design for the FRET reporter construct. The fluorescence lifetime of CFP was measured in Lenti-X 293T cells. “Donor only”: Lenti-X 293T cells expressing BTN3A1 with N-terminally fused CFP. “Donor + Acceptor”: cells co-expressing BTN3A1 with N-terminally fused CFP and BTN2A1 with N-terminally fused YFP. The *N* value is provided for each group (mean ± SEM). *P* values were calculated by the Kruskal‒Wallis test with Dunn’s multiple comparisons post-test. Zol, zoledronate. Y127A, the 3A2 complex variant harboring a BTN3A1-Y127A mutation to disrupt oligomeric assembly; AAR, the 3A2 complex variant harboring BTN3A1-H381R/BTN2A1-R477A/T510A mutations to abolish HMBPP binding. **e** FRET efficiency was determined in Lenti-X 293T cells expressing the 3A2 complex, AAR, and Y127A variants. The mean fluorescence lifetime in each “Donor only” group was used as a control to calculate FRET efficiency in each “Donor + Acceptor” group. The *N* value is provided for each group (mean ± SEM). *P* values were calculated using the Kruskal‒Wallis test with Dunn’s multiple comparisons post hoc test. **f** TIRF-SIM super-resolution imaging of NIH-3T3 cells expressing the WT 3A2 complex or its variants ( ± 10 μM zoledronate). Raw images were cropped to highlight regions of interest. CCC, the 3A2 complex variant harboring G130C in BTN2A1, D132C in BTN3A1, and the D132C mutation in BTN3A2, respectively, to lock the oligomeric state by intermolecular disulfide bonds. NIH-3T3 cells were used instead of Lenti-X 293T cells because of their superior adhesion properties, which improved the signal-to-noise ratio in TIRF–SIM imaging. Zol, zoledronate. **g** Violin plots of the length distribution for all filaments identified in the SIM images from **f**. *P* values were calculated using the Kruskal‒Wallis test with Dunn’s multiple comparisons post-test.

To investigate the role of pAgs on BTN molecules, we successfully obtained cryo-EM structures of the 3A2 complex and 3A3 complex in complex with HMBPP at overall resolutions of 3.0 Å and 3.8 Å, respectively (Supplementary Figs. S2, S3). In the presence of HMBPP, cryo-EM structures revealed that both 3A2 and 3A3 complexes adopt tetrameric architectures, each composed of a BTN2A1 homodimer paired with either a BTN3A1/3A2 or BTN3A1/3A3 heterodimer, arranged in a scissor-like conformation, as previously reported^28,29^ (Fig. 2a; Supplementary Fig. S4a). The assembly mechanisms of the pAg-bound 3A2 complex and the 3A3 complex are highly similar. The following structural analysis takes the 3A2 complex as an example. Structural analysis identified three critical interfaces for tetramer formation: (1) IgV–IgV domain interactions between BTN2A1 and BTN3A2; (2) extensive contacts within the TM and JM regions of the BTN2A1 homodimer, the BTN3A1/BTN3A2 heterodimer; and (3) B30.2–B30.2 domain interactions between BTN2A1 and BTN3A1, which are facilitated by HMBPP (Supplementary Fig. S4b–f). These interfaces are involved in a network of disulfide bonds, hydrogen bonds, cation–π interactions, and van der Waals forces (Supplementary Fig. S5), largely similar to those reported in recent studies^28,29^.

Compared with the crystal structure of the BTN2A1–BTN3A1 extracellular domain complex (PDB ID: 8DFX^33^), which can assemble into higher-order oligomers in the crystal lattice (Supplementary Fig. S6a), the extracellular domains of the HMBPP-bound 3A2 complex exhibit substantial conformational changes. The interchain angle between the extracellular domains of BTN2A1 and BTN3A2 in the HMBPP-bound 3A2 complex decreased by 7.1° (Supplementary Fig. S6b), whereas the dimerization angles of the extracellular domains increased by 3.5° for the BTN2A1 dimer and by 7.7° for the BTN3A1/BTN3A2 heterodimer (Supplementary Fig. S6c, d).

Docking the HMBPP-bound 3A2 tetramer structure into the higher-order oligomer, on the basis of the IgV_BTN2A1_–IgV_BTN3A1_ interface, revealed curvature in the extracellular region and steric clashes in the intracellular region (Fig. 2b). These observations suggest that in the absence of pAg, the JM and B30.2 domains are flexible. Upon binding to HMBPP, these domains become stabilized, leading to rigidification of the intracellular region. This induces steric hindrance within intracellular domains and a change in the transit curvature of the extracellular domains, thereby destabilizing higher-order oligomers and promoting their dissociation into discrete tetrameric 3A2 complexes (Supplementary Video S1).

To test the hypothesis that pAgs induce the dissociation of oligomers, we first assessed the effect of pAgs on the oligomerization state of the 3A2 complex in solution using AUC (Fig. 2c). Compared with that of the WT 3A2 complex in the absence of HMBPP, the proportion of HMWS, corresponding to molecular weights exceeding 500 kDa, was significantly reduced upon the addition of HMBPP. These results suggest that HMBPP induces the dissociation of higher-order BTN oligomers into tetrameric units of the 3A2 complex.

To investigate the oligomerization states of BTN molecules on the cell membrane, we employed fluorescence lifetime imaging microscopy combined with fluorescence resonance energy transfer (FLIM-FRET). CFP and YFP were fused to the N-termini of BTN3A1 and BTN2A1, respectively, and co-expressed with BTN3A2 (Fig. 2d; Supplementary Fig. S7a, b). Cells expressing the WT 3A2 complex exhibited a marked decrease in the CFP fluorescence lifetime, indicating close proximity among those tetrameric 3A2 units and suggesting higher-order oligomerization. In contrast, treatment with HMBPP or zoledronate abolished the change in CFP fluorescence lifetime, indicating dissociation of the higher-order BTN oligomers (Fig. 2d, e). As controls, we generated a 3A2 complex variant carrying the H381R mutation in BTN3A1 and the R477A/T510A mutations in BTN2A1 (referred to as AAR) to disrupt pAg binding^34^, or the Y127A mutation in BTN3A1 to disrupt higher-order oligomer formation (Fig. 1e). As expected, the FRET signal of the Y127A complex decreased, and the FRET signal of the AAR complex remained unchanged upon pAg treatment (Fig. 2d, e).

To further validate these findings, we used an alternative approach by fusing CFP or YFP to the C-terminus of BTN3A2 (Supplementary Fig. S7c, d). Consistent with previous results, cells expressing the WT 3A2 complex exhibited a strong FRET signal, which was eliminated by pAg treatment, suggesting that pAg induces the dissociation of higher-order BTN oligomers. In control experiments, the FRET signal was absent in cells expressing the Y127A complex, and the AAR complex remained unaffected by pAg treatment (Supplementary Fig. S7e, f). Collectively, these FRET data show that the 3A2 complex forms higher-order oligomers on the cell membrane, and that this oligomerization is disrupted upon pAg treatment.

To validate the model in which pAg induces the dissociation of BTN oligomers, we employed TIRF-SIM to monitor alterations in BTN clusters on the cell membrane after pAg treatment. For the WT 3A2 complex, pAg addition led to a significant decrease in the length of membrane-associated clusters (Fig. 2f, g). However, when BTN oligomers were locked by intermolecular disulfide bonds (CCC mutant, the 3A2 complex variant harboring G130C in BTN2A1, D132C in BTN3A1, and the D132C mutation in BTN3A2, respectively) or when pAg sensing was disrupted by AAR mutations, pAg was unable to induce cluster dissociation. Consistent with our model, pAg treatment did not affect the length of membrane-bound clusters in cells expressing the Y127A complex, in which the higher-order oligomers were already disrupted (Fig. 2f, g).

In summary, we demonstrate that pAg binding triggers the disassembly of BTN higher-order oligomers. To corroborate this model, we used the AUC to monitor the dissociation of BTN oligomers in solution upon pAg binding. Furthermore, we evaluated oligomer disassembly on the cell membrane using FLIM-FRET and TIRF-SIM, confirming a mechanism that is distinct from that of recently proposed models^28,29^.

### Vγ9Vδ2 TCR binds to the BTN tetramer independently of pAg

The 3A2 or 3A3 complex can associate with either the G115 TCR^9^ or MOP TCR^35^ in the presence of HMBPP, as shown by gel filtration analysis (Supplementary Fig. S8a–d). We determined the structures of the G115 TCR–3A2, MOP TCR–3A2, and G115 TCR–3A3 complexes at resolutions ranging from 3.3 to 3.8 Å in the presence of HMBPP (Supplementary Figs. S9, S10). These structural findings, which are consistent with recent studies^28,29^, reveal a widely accepted dual-ligand recognition mechanism. The G115 TCR binds to BTN ectodomains, with the Vγ9 chain interacting laterally with IgV_BTN2A1_, while the Vδ2 chain apical face engages IgV_BTN3A2_ (Supplementary Fig. S11a, b). A similar interaction pattern was observed in the MOP TCR–3A2 complex (Supplementary Fig. S11c). The detailed interactions between the BTN complex and the G115 TCR closely resembled those reported in recent studies^28,29^. Interestingly, across all the datasets of these structures, we consistently observed a small subset of particles in which another TCR bound exclusively to the BTN2A1 IgV domain, which is consistent with the findings of a recent report^28^ (Supplementary Fig. S12). The physiological relevance of the additional interface between BTN2A1 and TCRγ9 remains to be elucidated.

The recognition of the BTN complex by the Vγ9Vδ2 TCR in the G115 TCR–3A2 complex involves both somatically recombined complementarity-determining region 3 (CDR3) and germline-encoded regions such as CDR1, CDR2, and hypervariable 4 (HV4) (Supplementary Fig. S11d). This recognition mode is consistent with the results of prior functional studies showing the necessity of both CDRs and HV4 regions for BTN recognition by the Vγ9Vδ2 TCR^19,20,36,37^. Structural analysis revealed intricate interaction networks of IgV_BTN3A2_–Vδ2 and IgV_BTN2A1_–Vγ9 interfaces involving at least 18 hydrogen bonds, two cation–π interactions, and extensive van der Waals forces (Supplementary Figs. S11e, S13).

Our structures also provide a structural framework explaining decades of mutagenesis data related to pAg-induced activation of the Vγ9Vδ2 TCR^19,20,22,33,34,36–39^. Mapping these mutations onto the G115 TCR–3A2 structure revealed that all the previously identified critical mutations localize precisely to the interfaces between the G115 TCR and the 3A2 complex, as observed in our structures (Supplementary Fig. S11f). The striking concordance between structural and functional data underscores the physiological importance of our structures.

**Fig. 3:**
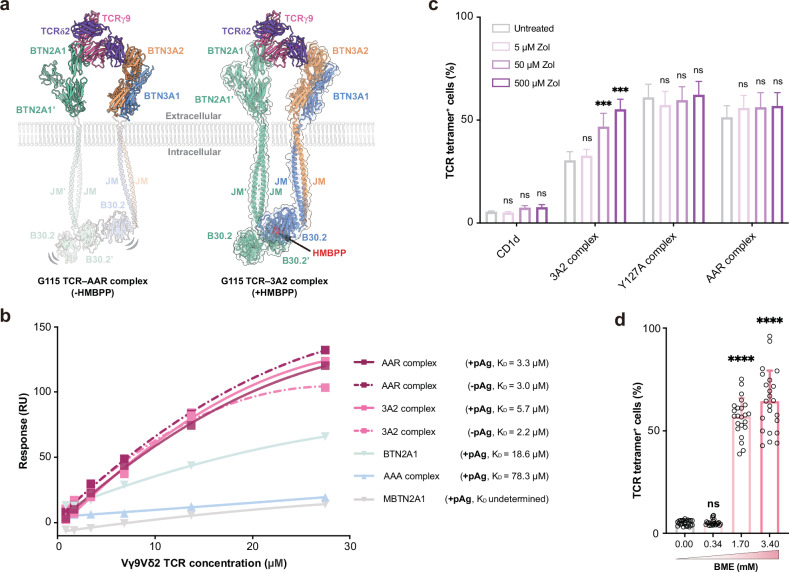
Vγ9Vδ2 TCR binding to the BTN tetramer is independent of pAgs. **a** Left: cryo-EM structure of the Vγ9Vδ2 TCR (G115 prototype) in complex with the ectodomain of the AAR complex in the absence of HMBPP. The variable domains of the TCR bind IgV_BTN2A1_ and IgV_BTN3A2_. Intracellular regions exhibit conformational flexibility (unresolved in the cryo-EM map) and are depicted as transparent cartoons. AAR complex, a 3A2 complex variant with mutations H381R in BTN3A1 and R477A/T510A in BTN2A1 B30.2 domains to disrupt pAg binding. Right: overall cryo-EM structure of the Vγ9Vδ2 TCR (G115 prototype) in complex with the HMBPP-bound WT 3A2 complex. The structure is presented in a cartoon representation, with the HMBPP highlighted in sphere representation in red. BTN molecules are displayed as transparent surfaces. Subunit rendering: BTN2A1 (medium aquamarine), BTN3A1 (cornflower blue), BTN3A2 (sandy brown), BTN3A3 (khaki), TCRδ (blue violet), and TCRγ (hot pink). The statistics for the model are shown in Supplementary Table S1. **b** Saturation binding curves of the G115 TCR measured by SPR. WT 3A2 and AAR complexes were tested with or without 4 μM HMBPP. WT or mutant BTN2A1 and the AAA complex were tested in the presence of only 4 μM HMBPP. AAA complex, a 3A2 complex variant carrying R124A/Y133A mutations in BTN2A1 and D132A in BTN3A2. MBTN2A1, a BTN2A1 variant carrying E63A/R65A/F71A/K79A/R84A/E87A/R131A/E135A mutations in BTN2A1 to disrupt TCR binding. RU, resonance units. *K*_d_, dissociation constant at equilibrium ± SEM. **c** G115 TCR-FITC tetramer staining of NIH-3T3 cells expressing the WT 3A2 complex or its variants after treatment with zoledronate. CD1d-expressing cells served as negative controls. The frequency of TCR tetramer^+^ cells was analyzed by flow cytometry. ns, no significance, ***P < 0.001, two-way ANOVA with Dunnett’s multiple comparison test. Zol, zoledronate. **d** G115 TCR-FITC tetramer staining of NIH-3T3 cells transfected with the CCC complex after they were incubated with the indicated concentrations of BME in the absence of pAgs. The frequency of TCR tetramer^+^ cells was analyzed by flow cytometry. ns, no significance, ****P < 0.0001, by the Kruskal‒Wallis test with Dunn’s multiple comparisons post-test. BME, β-mercaptoethanol.

To investigate the role of pAg in BTN complex recognition by the Vγ9Vδ2 TCR, we determined the cryo-EM structure of the G115 TCR–AAR complex at 3.2 Å resolution (Supplementary Figs. S8f, S14). The AAR complex is a 3A2 complex variant carrying the H381R mutation in BTN3A1 and the R477A/T510A mutations in BTN2A1 to disrupt pAg binding^34^. Unlike the structure of the HMBP-bound G115 TCR–3A2, the extracellular domains remain well resolved in the absence of HMBPP. However, the TMs, JMs, and B30.2 domains are invisible in the final reconstruction, indicating substantial conformational flexibility in these regions (Fig. 3a). Structural superposition revealed that the overall extracellular architecture was similar, with a root mean standard deviation (RMSD) of 0.3 Å, indicating that the TCR–BTN interface remained unchanged.

Our structural data suggest that pAg does not affect the interaction between the Vγ9Vδ2 TCR and the BTN complex. To test this hypothesis, we measured binding affinities by surface plasmon resonance (SPR) (Fig. 3b; Supplementary Fig. S15). Purified tetrameric WT and AAR complexes showed similar binding affinities for the G115 TCR in the presence of pAg, with dissociation constants (*K*_d_) of 5.7 μM and 3.3 μM, respectively. In the absence of pAg, the binding affinity was also similar, with *K*_d_ values of 2.2 μM and 3.0 μM for the respective complexes. Although cryo-EM analysis revealed an intricate interaction network between the TCRδ chain and the BTN3A2 IgV domain, SPR analysis did not reveal a measurable binding affinity between the TCR and the BTN3A1/3A2 complex, which is consistent with previous findings^40^. The binding of the BTN2A1 dimer was weaker, with a *K*_d_ value of 18.6 μM, which is comparable to that reported previously^19,20^. In contrast, the AAA complex and the MBTN2A1 dimer mutants designed to disrupt TCR binding exhibited markedly reduced binding affinities, with a *K*_d_ value of 78.3 μM for the AAA complex (a 3A2 complex variant carrying R124A/Y133A mutations in BTN2A1 and D132A in BTN3A2) and no detectable binding for MBTN2A1 (a BTN2A1 variant carrying E63A/R65A/F71A/K79A/R84A/E87A/R131A/E135A mutations in BTN2A1) (Fig. 3b; Supplementary Fig. S15). These results demonstrate that, similar to the tetrameric 3A2 complex, the G115 TCR interacts with the ectodomains of BTN2A1 and BTN3A2 independent of the binding of HMBPP to the B30.2 domain.

### PAg drives Vγ9Vδ2 TCR binding to BTN molecules on the cell membrane

To further elucidate the role of pAgs, we performed streptavidin-FITC-conjugated Vγ9Vδ2 TCR tetramer staining assays to evaluate how pAgs affect Vγ9Vδ2 TCR binding to BTN molecules on the cell membrane (Supplementary Fig. S16). In cells expressing the 3A2 complex, substantial G115 TCR staining was detected in the absence of zoledronate treatment. Increasing concentrations of zoledronate markedly enhanced G115 TCR binding in these cells (Fig. 3c). In contrast, cells expressing the AAR complex, whose pAg binding is impaired, showed no increase in G115 TCR staining following zoledronate treatment. Strikingly, cells expressing the Y127A complex, which forms a constitutive tetramer, displayed high levels of G115 TCR binding that were not further increased by zoledronate (Fig. 3c). These results suggest that the dissociation of the oligomeric 3A2 complex into tetrameric units is critical for augmenting TCR binding.

To verify that oligomer dissociation is necessary for pAg-induced TCR binding, we performed Vγ9Vδ2 TCR tetramer staining in cells expressing the CCC variant, which contains the G130C, D132C, and D132C mutations in BTN2A1, BTN3A1, and BTN3A2, respectively. These mutations were designed to stabilize the oligomeric state through the formation of intermolecular disulfide bonds. In the absence of β-mercaptoethanol (BME), Vγ9Vδ2 TCR tetramers failed to stain cells expressing the CCC complex (Fig. 3d). By contrast, treatment with BME concentrations at 1.70 mM or higher induced a marked increase in Vγ9Vδ2 TCR tetramer binding. These data confirm that pAg-induced TCR binding on the cell membrane necessitates the dissociation of 3A2 complex oligomers (Fig. 3d).

In summary, these findings demonstrate that the tetrameric BTN complex directly interacts with the TCR. Notably, pAg does not affect TCR binding affinity in this tetrameric configuration. In contrast, higher-order BTN oligomers inhibit TCR binding. pAgs promote BTN–TCR interactions by dissociating higher-order oligomers into competent tetrameric units, in a mechanism distinct from those recently reported^28,29^.

### Higher-order BTN oligomer dissociation contributes to pAg-dependent γδ T-cell activation

Combining AUC, TIRF, and FLIM–FRET, we have showed that pAg induces the dissociation of higher-order BTN oligomers into tetramers. To test whether this dissociation is required for γδ T-cell activation, we compared cells expressing the 3A2, Y127A, or AAR BTN complexes in the presence or absence of pAgs. The 3A2 complex formed higher-order oligomers that dissociated into tetramers upon pAg stimulation. The AAR complex failed to bind pAg and remained oligomeric, whereas the Y127A complex existed constitutively as a tetramer, independent of pAg.

**Fig. 4:**
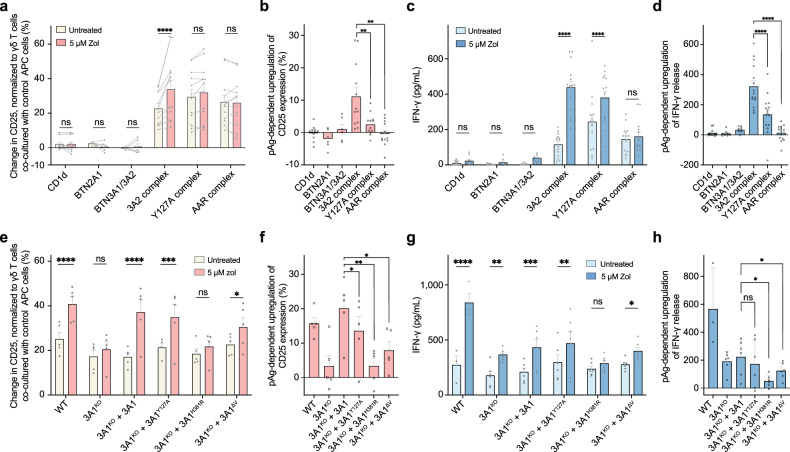
PAg responses of Vγ9Vδ2 T cells are mediated by the dissociation of BTN higher-order oligomers. **a**, **b** CD25 expression levels in Vγ9Vδ2 T cells co-cultured with NIH-3T3 fibroblasts expressing different BTN molecules pretreated with or without 5 μM zoledronate (**a**) and the zoledronate-induced upregulation of CD25 expression in Vγ9Vδ2 T cells (**b**). CD1d-expressing cells served as negative controls. Zol, zoledronate. ns, no significance, ****P < 0.0001, by two-way ANOVA with Sidak’s multiple correction test in **a**. **c**, **d** IFN-γ concentration in the Vγ9Vδ2 T-cell culture medium (**c**) and changes in the IFN-γ concentration in the culture medium of Vγ9Vδ2 T cells before and after zoledronate treatment (**d**). Zol, zoledronate. ns, no significance, ****P < 0.0001, by two-way ANOVA with Sidak’s multiple correction test in **c**. **e**, **f** CD25 upregulation in Vγ9Vδ2 T cells following co-culture with K562 *BTN3A1*^*–/–*^ cells reconstituted with WT or mutant BTN3A1. Vγ9Vδ2 T cells were co-cultured with K562 cells lacking endogenous BTN3A1 that were transduced to express either WT BTN3A1 or the indicated mutants, followed by pretreatment with 5 μM zoledronate. Surface CD25 expression on Vγ9Vδ2 T cells was measured by flow cytometry. Zol, zoledronate. **g**, **h** IFN-γ secretion by Vγ9Vδ2 T cells co-cultured with K562 *BTN3A1*^*–/–*^ cells reconstituted with WT or mutant BTN3A1. Changes in the IFN-γ concentration in the culture supernatant (**g**) and zoledronate-induced changes in the IFN-γ concentration relative to those in the untreated condition (**h**). Statistical significance was determined by two-way ANOVA followed by Fisher’s LSD test (ns, no significance, *P < 0.05, **P < 0.01, ***P < 0.001, ****P < 0.0001) in **e** and **g**. The data were subtracted from those of Vγ9Vδ2 T cells co-cultured with the NIH-3T3 cells in **g**. Zol, zoledronate.

We co-cultured primary Vγ9Vδ2 T cells expanded from human peripheral blood mononuclear cells (PBMCs) with NIH-3T3 cells expressing these complexes at comparable surface levels (Supplementary Fig. S17a, b). Consistent with previous reports^19,20^, the expression of BTN2A1 or BTN3A1/BTN3A2 alone did not induce CD25 upregulation or IFN-γ secretion in Vγ9Vδ2 T cells, regardless of pAg treatment (Fig. 4a, b; Supplementary Fig. S17c). In contrast, cells expressing the 3A2 complex induced robust CD25 upregulation, which was further augmented by zoledronate, indicating strong pAg-dependent activation (Fig. 4a, b). Moreover, pAg-induced CD25 upregulation was abrogated in Vγ9Vδ2 T cells co-cultured with cells expressing the AAR complex, which is also consistent with previous reports^40^.

We next examined the role of the BTN2A1–BTN3A1 interface using mutagenesis. pAg-dependent CD25 upregulation was abrogated in Vγ9Vδ2 T cells co-cultured with cells expressing the Y127A complex, indicating that this interface is required for pAg-dependent Vγ9Vδ2 T-cell activation. A similar pattern was observed for IFN-γ secretion; pAg-dependent IFN-γ release was robust in T cells co-cultured with 3A2 complex-expressing cells, but significantly reduced in those cocultured with the Y127A complex (Fig. 4c, d). These results suggest a critical role for the higher-order oligomer interface in pAg-dependent activation.

To validate these findings in a more physiological context, we extended the analysis to human K562 cells. We first generated a BTN3A1-deficient K562 cell line. As expected, WT K562 cells induced robust CD25 upregulation upon pAg treatment, whereas BTN3A1 deletion abrogated this response when co-cultured with primary Vγ9Vδ2 T cells (Fig. 4e, f). Reconstitution with WT or mutant BTN3A1 (Y127A, H381R, and ΔV, which truncates the IgV-like domain) revealed that H381R, which disrupts pAg binding, completely abrogated pAg-induced Vγ9Vδ2 T-cell activation. By contrast, Y127A and ΔV, which disrupt the BTN2A1–BTN3A1 oligomerization interface, significantly reduced CD25 upregulation compared to WT BTN3A1 (Fig. 4e, f). IFN-γ secretion by Vγ9Vδ2 T cells co-cultured with K562^3A1KO^ cells reconstituted with WT or mutant BTN3A1 (Y127A, H381R, and ΔV) generally paralleled the CD25 upregulation profile, but with a much smaller dynamic range (Fig. 4g, h). Notably, pAg-induced IFN-γ expression remained detectable in K562^3A1KO^ cells, indicating the presence of additional BTN-independent inputs or accessory ligands in this human context. Nonetheless, most of these results support that the BTN2A1–BTN3A1 interface is required for efficient pAg-dependent signaling in human cells.

In summary, results from both murine and human systems demonstrate that the dissociation of higher-order BTN oligomers markedly enhance pAg-dependent Vγ9Vδ2 T-cell activation. These findings support a model whereby the pAg-induced dissociation of higher-order BTN oligomers into discrete tetramers is required for γδ T-cell activation.

### In situ validation of pAg-induced dissociation of higher-order BTN oligomers

**Fig. 5:**
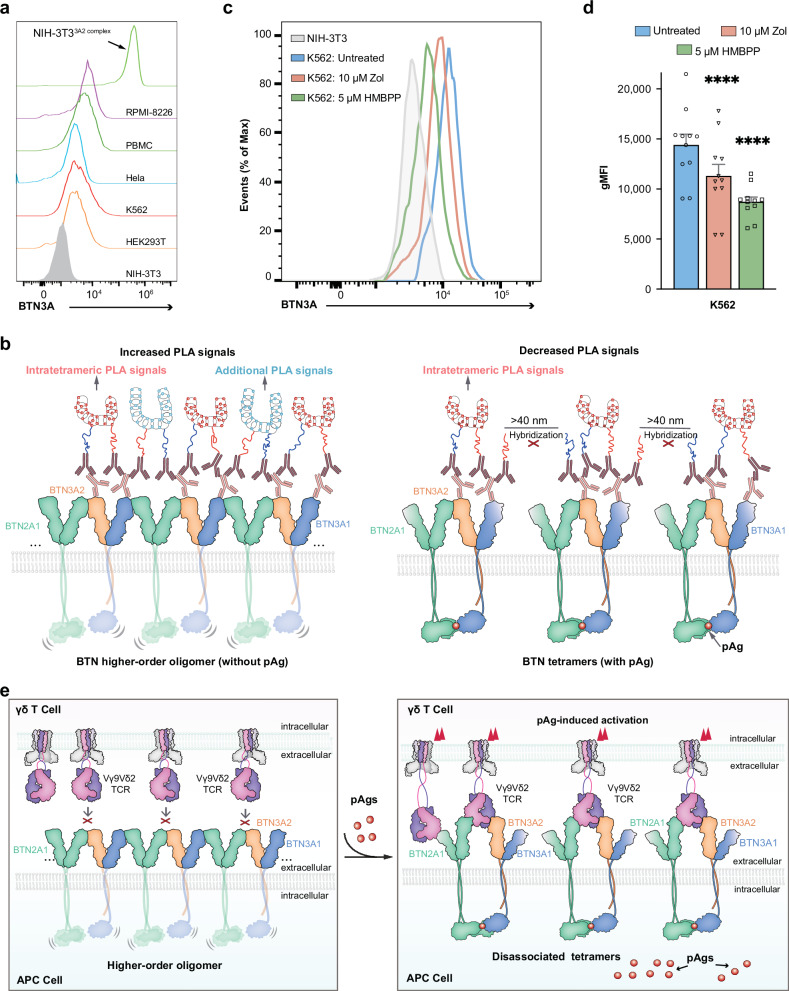
In situ validation of pAg-induced BTN higher-order oligomer disassembly. **a** Comparison of BTN3A expression levels between tumor cell lines and BTN3A-overexpressing NIH-3T3 cells. **b** Schematic illustration of in situ Duolink® PLA validation. In higher-order oligomeric BTN clusters, BTN3A protomers from adjacent tetramers are positioned within ~40 nm, contributing to increased PLA signals (left). Upon pAg-induced dissociation, the spatial separation of individual tetramers exceeds 40 nm, confining PLA signals to the BTN intratetramer (right). **c** Flow cytometry histograms of the PLA signal in NIH-3T3 (control) and K562 cells (untreated, 10 μM zoledronate, and 5 μM HMBPP). Zol, zoledronate. **d** Bar graph displaying the gMFI of the PLA signal in K562 cells under three conditions: untreated, 10 μM zoledronate and 5 μM HMBPP. The data are presented as the mean ± SEM. Zol, zoledronate. ****P < 0.0001, one-way ANOVA followed by Fisher’s LSD test. **e** Proposed model of pAg-mediated activation of the Vγ9Vδ2 TCR. In the absence of pAgs, BTN molecules form oligomers on the cell membrane with flexible intracellular regions (left). Upon pAg binding, the bending of extracellular regions and steric hindrance within the intracellular rigid segments of BTN molecules collectively trigger the dissociation of BTN oligomers into tetramers. The Vγ9Vδ2 TCR then engages the ectodomains of the tetrameric BTN complex (right).

To determine whether the higher-order oligomerization of BTN complexes arises from overexpression, we quantified the endogenous BTN3A levels in HEK293T, K562, HeLa, RPMI8226 cells and PBMCs using anti-BTN3A antibodies. In all cases, endogenous BTN3A levels were markedly lower than those in BTN3A-overexpressing NIH-3T3 cells (Fig. 5a), highlighting the importance of examining this mechanism in human cell lines.

We initially attempted in situ assessment of BTN oligomers using FRET and TIRF-SIM with BTN3A-targeting antibodies. However, the low abundance of endogenous BTN3A yielded insufficient signal for reliable analysis. We therefore employed the highly sensitive Duolink® proximity ligation assay (PLA) in K562 cells, which express moderate levels of BTN3A (Fig. 5a). PLA detects protein assemblies within ~40 nm proximity range. Compared with isolated BTN tetramers, higher-order BTN oligomers, formed by clustering multiple tetramers, are expected to generate stronger PLA signals because of more efficient ligation-dependent DNA circle formation and amplification (Fig. 5b).

In the absence of pAgs, K562 cells exhibited strong PLA signals, reflecting extensive BTN3A clustering. Upon pAgs stimulation, the PLA signals were markedly reduced, suggesting that clustered BTN3A dissociates in response to pAgs treatment (Fig. 5c, d). These in situ PLA results indicate that pAg induces the disassembly of higher-order BTN oligomers, which is consistent with our previously proposed model. Furthermore, this mechanism operates not only in BTN-transduced rodent cells but also in human cell lines, highlighting its physiological relevance.

## Discussion

Our integrated biophysical, biochemical, and cellular analyses support an unprecedented pAg-dependent γδ T-cell activation model, which is distinct from those recently reported^28,29^. In this model, BTN molecules form higher-order oligomers on the cell membrane in the absence of pAgs. Within these oligomer assemblies, the ectodomains adopt a tightly packed, side-by-side arrangement, whereas the cytoplasmic regions remain flexible to avoid steric hindrance. This compact ectodomain organization occludes the TCR–BTN binding interface, preventing γδ TCR recognition (Fig. 5e). Upon the intracellular binding of pAgs, these oligomers dissociate into discrete tetramers, enabling γδ TCR engagement (Fig. 5e).

The binding of pAg induces transient steric clashes at the intracellular domains of the BTN oligomer. In the intracellular regions, pAg functions as a molecular glue, enhancing the interaction between the B30.2 domains of BTN2A1 and BTN3A1 within the higher-order BTN oligomer. This interaction stabilizes the transmembrane and JM regions, creating steric clashes between adjacent BTN molecules and likely promoting transient bending of their extracellular domains, thereby driving the release of discrete BTN tetramers. Once liberated from higher-order oligomers, these tetramers adopt the characteristic “scissor-like” conformation and engage Vγ9Vδ2 TCRs in a dual-ligand mode, with detailed interactions consistent with those described in previous reports^28,29^, ultimately initiating γδ T-cell activation (Fig. 5e).

Recently, two models have been proposed for pAg-mediated γδ T-cell activation^28,29^. While the Vγ9Vδ2 TCR-bound conformation of BTN tetramers in our structure closely aligns with the dual-ligand binding mode described in these reports, our study reveals an earlier regulatory step. We demonstrate that BTN molecules pre-exist as higher-order oligomers on the cell surface and that pAg stimulation triggers their dissociation into discrete tetramers. This higher-order oligomer-to-tetramer transition defines the initiation phase of BTN–TCR signaling by controlling the remodeling of the oligomeric state of BTN complex. Therefore, we propose a unified model in which pAgs act in a stepwise manner: first, pAgs induce the dissociation of higher-order BTN oligomers into tetramers; subsequently, these tetramers adopt the characteristic “scissor-like” conformation and engage the Vγ9Vδ2 TCR through the dual-ligand binding mode. Our findings complement existing models by suggesting that pAg functions at an earlier stage to regulate BTN assembly dynamics prior to TCR engagement (Fig. 5e).

We noted an unexpected Vγ9Vδ2 TCR staining pattern for the AAR complex (Fig. 3c). Structural analysis suggested that the R447A/T510A substitutions in the BTN2A1 B30.2 domain weaken the interactions between cytosolic BTN2A1 and BTN3A1 within the BTN complex, thereby reducing the stability of higher-order oligomers. Although the AAR variant can still assemble into higher-order structures at the cell surface, its reduced stability likely permits transient exposure of the extracellular BTN2A1–BTN3A interface, enabling spontaneous Vγ9Vδ2 TCR engagement in the absence of pAg. Consistent with this scenario, AAR-expressing cells exhibited stronger TCR tetramer staining than WT 3A2-expressing cells without pAg stimulation do (Fig. 3c). We also note that the staining background (nonspecific tetramer binding) may differ among NIH-3T3 cells expressing distinct BTN complexes, which could complicate interpretation. Although the dual-ligand recognition mode has emerged as the prevalent model in the field, our cryo-EM structures confirm that the Vγ9Vδ2 TCR can bind the BTN2A1 homodimer independently of BTN3A2/BTN3A3 (Supplementary Fig. S12), which is consistent with the strong tetramer staining signals observed in cells expressing BTN2A1 alone^19,20,33^.

In the absence of pAg, the Y127A complex existed predominantly as tetramers, whereas the WT 3A2 complex formed higher-order oligomers that may occlude the Vγ9Vδ2 TCR binding interface. Therefore, cells expressing the Y127A complex displayed increased accessibility to the Vγ9Vδ2 TCR and enhanced basal activation. In line with this interpretation, Vγ9Vδ2 TCR tetramer staining was markedly stronger in Y127A-expressing cells than in WT 3A2-expressing cells in the absence of pAgs (Fig. 3c); Vγ9Vδ2 T cells co-cultured with either Y127A-expressing murine cells or Y127A-expressing human K562 cells exhibited modestly elevated basal CD25 expression and IFN-γ secretion (Fig. 4). These data support the model that pAg-induced dissociation of oligomeric BTN assemblies serves as a key switch to unmask the TCR-binding interface and initiate downstream signaling.

Prior studies have shown that the E63A^28,29,34^ mutation in BTN2A1, as well as the R73A^38^ or Y127A^38^ mutations in BTN3A1, all of which disrupt extracellular BTN2A1–BTN3A1 interactions (Supplementary Fig. S4b), markedly enhance the stimulation of Vγ9Vδ2 T cells. Among these mutations in BTN3A1, the Y127A or R73A mutation results in the greatest increase in HMBPP-induced TCR activation^38^. This enhanced Vγ9Vδ2 T-cell activation may be attributed to the dissociation of higher-order oligomers caused by these mutations that disrupt the BTN2A1–BTN3A1 interface within the oligomer.

A key physiological implication of our model is how inadvertent γδ T-cell activation is avoided. Endogenous IPP requires relatively high concentrations (EC₅₀ ≈ 36 μM; effective range 10–100 μM) to activate Vγ9Vδ2 T cells, whereas the microbe-derived pAg HMBPP triggers activation at sub-nanomolar levels (EC₅₀ ≈ 0.00051 μM), representing a > 10^4^-fold difference in potency^41^. This disparity is embedded in the oligomer–tetramer transition of BTN complexes. Under steady-state conditions, IPP remains below the threshold required to drive oligomer dissociation, maintaining BTN in a self-inhibited oligomeric state that occludes the Vγ9Vδ2 TCR-binding interface. Only when IPP accumulates sufficiently does it trigger the transition to tetramers, enabling TCR engagement and signaling. In contrast, microbial phosphoantigens efficiently induce this switch at low concentrations, allowing the rapid detection of infection. Thus, the oligomer–tetramer transition acts as a concentration-dependent molecular switch that prevents spurious activation while preserving sensitivity to microbial ligands.

These structural and functional insights not only reshape our understanding of γδ T-cell activation but also open new avenues for clinical immunotherapy. Researchers may develop more stable, tumor-targeting pAg prodrugs as novel cancer immunotherapeutic agents^41–43^ that activate Vγ9Vδ2 T cells by triggering the dissociation of BTN oligomers. In parallel, novel T-cell engagers (TCEs) can be designed to mimic the dual-ligand mode of BTN-mediated TCR engagement^44^. By engineering their antibody domains to recognize specific tumor-associated antigens, these TCEs can precisely redirect Vγ9Vδ2 T cells against diverse tumor types, providing a promising approach for precision cancer immunotherapy.

Notably, pAg-mediated Vγ9Vδ2 T-cell activation is highly complex and displays marked species- and cellular context-dependent differences. The comparison with a previous study is informative^22^. In the HEK293T BTN3A-deficient system used in the previous work, no pAg-independent activation was detected, and deletion of the BTN3A1 IgV domain (3A1^ΔV^) also abolished responsiveness. However, in our K562 system, 3A1^ΔV^ similarly abrogated pAg-induced activation, but the basal activity persisted (Fig. 4c). A possible explanation may be that different cell types harbor distinct accessory pathways or co-stimulatory inputs that can partially bypass canonical BTN-dependent signaling to varying extents^45,46^. For example, Vγ9Vδ2 T cells are known to express natural killer cell receptors (NKRs)^47^, and variations in NKR ligand expression across antigen-presenting cells may further contribute to the observed differences in functional output.

In addition, differences between rodent and human systems have been well documented^19–22,33,40,48–50^. Rodent cells engineered to express BTN3A1 and BTN2A1 are sufficient to induce robust Vγ9Vδ2 T-cell activation^19–21,33^, and the co-expression of BTN3A2 slightly enhances the activation^19^. In contrast, human cells display more stringent and context-dependent requirements for BTN composition and coordination; co-expression of BTN3A1 with BTN3A2 (or BTN3A3) markedly enhances pAg responses compared with BTN3A1 alone^22,23^. Similarly, responses to agonistic antibodies vary across systems: BTN3A1-expressing CHO cells support activation in the absence of BTN2A1^50^, and murine NXS2 cells expressing human BTN3A1 alone do not recapitulate this responsiveness to 20.1^40^, whereas BTN2A1 is required in HEK293T cells^19–21^. These observations indicate that BTN-mediated signaling is strongly influenced by the cellular environment.

In conclusion, our study reveals a pAg-dependent mechanism of γδ T-cell activation. Across diverse cellular systems, BTN oligomerization and pAg-induced dissociation emerge as a conserved structural feature underlying TCR engagement. The amplitude and basal tone of the response are influenced by cell type and other specific factors, and further validation in additional systems is warranted. Nonetheless, the fundamental biochemical properties of BTN complexes are likely conserved, highlighting a mechanistic principle that may inform the design of next-generation γδ T-cell-based immunotherapies.

## Materials and methods

### Construct design

The cDNAs encoding human BTN2A1, BTN3A1, BTN3A2, and BTN3A3 were codon-optimized and synthesized by Tsingke Biotechnology Co., Ltd. These cDNAs were subsequently subcloned and inserted into an optimized pCAG vector. A synthetic signal peptide (MDMRVPAQLLGLLLLWLSGARC)^51^ was fused to the N-terminus of BTN2A1, BTN3A1, BTN3A2, and BTN3A3. To facilitate protein purification, a Flag tag (MDYKDDDDK) was inserted at the C-terminus of BTN2A1 or BTN3A3, while a twin-strep II tag (WSHPQFEKGGGSGGGSGGSAWSHPQFEK) was added to the C-terminus of BTN3A1. The coding sequences for the extracellular domains of the G115^9^ and MOP^35^ TCRδ2 and TCRγ9 chains were also codon-optimized and fused with the same synthetic signal peptide at their N-termini. Additionally, a 6× His tag and a cFos tag^52^ (ASTDTLQAETDQLEDEKYALQTEIANLLKEKEKLGAP) were inserted at the C-terminus of TCRδ2. The JunW_ph1_ tag^52^ (ASAAELEERVKTLKAEIYELRSKANMLREQIAQLGAP) was fused at the C-terminus of TCRγ9. Detailed information regarding these constructs can be found in the Supplementary Information.

### Protein purification of the 3A2 complex and 3A3 complex

To purify the 3A2 complex, 2.4 mg of plasmids encoding BTN2A1, BTN3A1, and BTN3A2 (0.8 mg per plasmid) were pre-incubated with 5 mg of 40 K polyethyleneimine (PEI) (Yeasen) in 40 mL of fresh medium (Sino Biological Inc.) for 30 min, as previously described^53–55^. The plasmid-PEI mixture was then transfected into 0.8 L of ExpiHEK293F cells (Thermo Fisher Scientific, Inc.). The transfected cells were cultured for 3 days at 37 °C with 5% CO_2_ in a Multitron-Pro shaker (Infors, 120 rpm) before collection. The harvested cells were resuspended in lysis buffer containing 25 mM HEPES, pH 7.4, 150 mM NaCl, 2 μg/mL pepstatin, 1 μg/mL aprotinin, and 2 μg/mL leupeptin. The cell membranes were solubilized overnight at 4 °C using 1% (w/v) lauryl maltose neopentyl glycol (LMNG, Anatrace) and 0.1% cholesteryl hemisuccinate tris salt (CHS, Anatrace). The cell lysate was centrifuged at 12,500 rpm for 1 h to remove insoluble materials. The supernatant was added to anti-Flag M2 affinity resin (Millipore Sigma), which was subsequently washed with wash buffer (25 mM HEPES, pH 7.4, 150 mM NaCl, and 0.02% (w/v) GDN (glyco-diosgenin; Anatrace)) and eluted using wash buffer supplemented with 400 μg/mL Flag peptide. The eluent from the anti-Flag resin was loaded onto Strep-Tactin XT resin (IBA), washed with wash buffer, and eluted using a buffer containing 25 mM HEPES, pH 7.4, 150 mM NaCl, 0.02% (w/v) GDN, and 50 mM d-biotin. The 3A2 complex was concentrated to 0.8 mL and further purified using size-exclusion chromatography (SEC) on a Superose 6 Increase 10/300 column (GE Healthcare). Western blot analysis was performed on SEC fractions using mouse monoclonal antibodies against the Flag tag (CW0287M, CWBIO) and Strep tag (ab76949, Abcam) for verification. The 3A3 complex was purified using similar procedures.

### Preparation of soluble Vγ9Vδ2 TCR

Plasmids encoding the soluble TCRγ9 and TCRδ2 chains (1 mg per plasmid) were pre-mixed with 4 mg of PEI in 45 mL of fresh medium for 20 min. This mixture was then transfected into ExpiHEK293F cells. The transfected cells were cultured at 37 °C under 5% CO_2_ for 5 days before being harvested. After centrifugation at 4000× *g* for 20 min, the supernatant was collected and filtered through a 0.45 μm membrane to remove cell debris. The filtered medium was concentrated to ~150 mL and loaded onto Ni-NTA resin (Qiagen). The resin was extensively washed with buffer containing 25 mM HEPES, pH 7.4, 150 mM NaCl, and 30 mM imidazole. The secreted Vγ9Vδ2 TCR was eluted using wash buffer supplemented with 300 mM imidazole. The eluate was concentrated and further purified by SEC on a Superdex 200 Increase 10/300 GL column in buffer containing 25 mM HEPES, pH 7.4 and 150 mM NaCl.

### Reconstitution of G115 and MOP TCR with the 3A2 complex or 3A3 complex

The WT or mutant BTN complexes were incubated with an excess molar ratio of the Vγ9Vδ2 TCR to facilitate the formation of a stable complex. This incubation was performed at 4 °C for 1 h with 40 µM HMBPP. The mixture was concentrated to a final volume of 0.8 mL. Any excess TCR was subsequently removed using SEC (Superose 6 increase 10/300, GE Healthcare) in buffer containing 25 mM HEPES, pH 7.4, 150 mM NaCl, 0.02% (w/v) GDN, and 4 µM HMBPP. Finally, the purified TCR–BTN complex was concentrated, and prepared for cryo-EM samples.

### Cryo-EM sample preparation and data collection

Holey carbon grids (Quantifoil, Au, 300-mesh, R1.2/1.3) were glow-discharged at 15 mA for 30 s. A 3 μL aliquot of concentrated sample was applied to the grid and blotted for 3 s under 100% humidity at 8 °C. The grid was then rapidly plunged into liquid ethane using a Vitrobot Mark IV (Thermo Fisher Scientific).

For the cryo-sample of the 3A2 complex with HMBPP, the grids were transferred to a Krios electron microscope (Thermo Fisher Scientific) operating at 300 kV and equipped with a Falcon 4i direct electron detector and a Selectris X energy filter. Movies in electron event representation (EER) format were automatically collected using an EPU (Thermo Fisher Scientific) with a slit width of 10 eV and a defocus value ranging from −1.0 to −2.0 µm with a nominal magnification of 165,000×^56^, corresponding to a physical pixel size of 0.72 Å per pixel. Each movie was recorded for 4.98 s with a total dose of 50 e^−^ per Å^2^ contained in 1530 EER frames.

For other cryo-samples, the grids were transferred to a Krios electron microscope (Thermo Fisher Scientific) operating at 300 kV and equipped with a Gatan K3 Summit detector and GIF Quantum energy filter. Movie stacks were automatically collected using EPU (Thermo Fisher Scientific) with a preset defocus value ranging from −1.0 to −2.0 µm in super-resolution mode with a nominal magnification of 81,000×^56^, corresponding to a physical pixel size of 1.087 Å per pixel or 1.0773 Å per pixel. Each movie was recorded for 2.56 s with a total dose of 50 e^−^ per Å^2^ contained in 32 frames.

### Cryo-EM data processing

The data processing flowchart for the dataset of 3A2 complex oligomers is presented in Supplementary Fig. S1e. Movie stacks were motion-corrected using MotionCor2 v1.2.6^57^, and the dose-weighted micrographs were subjected to Patch CTF Estimation in cryoSPARC v4^58^. The template-picking particles were extracted and binned by a factor of 4. The particles were subjected to multiple rounds of 2D classification to remove contaminations and junk particles. Cleaned particles were recentered and re-extracted and binned by a factor of 2. After several rounds of 2D classification, the selected particles were subjected to ab initio reconstruction (K = 5). Particles from the best classes were combined and refined by non-uniform refinement^59^, yielding an oligomer map at 11.36 Å global resolution. The refined particles were not unsampled and further refined, as no discernible improvement in resolution or map quality was achieved. The validation and analysis of the density maps can be found in Supplementary Fig. S1f, g.

A flowchart for the data processing of the 3A2 complex with HMBPP is presented in Supplementary Fig. S2c. EER movies were subjected to Patch Motion Correction and Patch CTF Estimation in cryoSPARC v4^58^. Micrographs exhibiting a CTF estimated resolution of less than 4 Å, an astigmatism greater than 500, and a relative ice thickness greater than 1.1 were discarded by the Manually Curate Exposure job. To perform template-based particle picking, a subset of motion-corrected micrographs was initially processed to generate 2D class averages with clear protein features. These class averages were subsequently utilized as references for template picking, which was conducted on the complete dataset. The extracted particles were initially binned by a factor of 4 and subjected to multiple rounds of 2D classification to discard junk particles. Cleaned particles were recentered and re-extracted without downscaling and subjected to parallel ab initio reconstruction (K = 5). Particles from the best classes were merged, the duplicates were removed, and the resulting particles were refined using the best initial model as the reference. The refined particles were subsequently subjected to rounds of heterogeneous three-dimensional (3D) classification, local motion correction, and non-uniform refinement^59^, yielding a 2.96 Å overall reconstruction. The resulting particles were subjected to local refinement with a soft mask that encompassed the extracellular domain or the micelle plus the intracellular domain, yielding local maps at 2.90 Å resolution and 2.73 Å resolution, respectively. The validation and analysis of the density maps can be found in Supplementary Fig. S3a–g.

The data processing flowchart for the dataset of the 3A3 complex with HMBPP is presented in Supplementary Fig. S2d. Movie stacks were motion-corrected using MotionCor2 v1.2.6^57^, and dose-weighted micrographs were imported into cryoSPARC v4^58^. Following the aforementioned data processing strategies, non-uniform refinement^59^ generated a 3.86 Å overall reconstruction. After that, those poses were transferred to RELION-5^60^ (https://github.com/3dem/relion/tree/ver5.0) using csparc2star.py in the UCSF pyem program packages^61^ and then subjected to Bayesian polishing^62^. Bayesian polished particles were subjected to Relion 3D auto-refinement, yielding an overall density map with Fourier shell correlation (FSC) resolution of 3.81 Å, which exhibited map density superior to that of the map generated by cryoSPARC^58^. Subsequent consensus refinement with a soft mask that encompassed the extracellular domain or the micelle plus intracellular domain yielded two local maps at 3.47 Å resolution and 3.48 Å resolution, respectively. To improve the density of the B30.2 domain of BTN3A3, the resulting intracellular domain-refined particles were subjected to several rounds of focused 3D classification (skip alignment) with a sphere mask encompassing the targeted region. Subsequent consensus refinement of the sorted particles yielded a local density map at 3.26 Å resolution with superior B30.2 densities of BTN3A3 (Supplementary Fig. S2d). Validations and analyses of the density maps can be found in Supplementary Fig. S3h–l. A similar data processing strategy was applied to the datasets of the G115 TCR–3A2 complex (Supplementary Fig. S9d), MOP TCR–3A2 complex (Supplementary Fig. S9e), and G115 TCR–3A3 complex (Supplementary Fig. S9f), with the addition of HMBPP.

To determine the density of the TCR only bound to the other side of the BTN2A1 IgV domain within the G115 TCR–3A2 complex, we first generated a spherical mask centered on the interaction interface between the TCR and BTN2A1. This mask was subsequently applied to multiple rounds of 3D classification in RELION^60^. In each round, the particles of good classes were merged and deduplicated from the last four iterations. The selected particles were then imported into cryoSPARC^58^ for homogeneous reconstruction followed by non-uniform refinement, yielding an overall density map at ~3.8 Å resolution. In this map, one TCR engaged both BTN2A1 and BTN3A2, while the other TCR bound only to the BTN2A1 IgV domain. To further improve the density of the TCR bound to the BTN2A1 IgV domain, the particles were re-centered to the BTN2A1–TCR interface and re-extracted. Subsequent local refinement with a protein mask produced a local density map with a resolution of 4.0 Å. To improve the resolution further, the corresponding particles were transferred back to RELION^60^ for multiple rounds of 3D classification (skip align) with a protein mask to remove poorly aligned particles and junk. The cleaned particles were then subjected to consensus refinement with a protein mask, which improved the resolution to 3.8 Å. A similar data processing strategy was applied to the dataset of the G115 TCR–3A3 complex, with the addition of HMBPP.

For the dataset of the G115 TCR–AAR complex, the flowchart for data processing is presented in Supplementary Fig. S14b. Movie stacks were motion-corrected using MotionCor2 v1.2.6^57^, and dose-weighted micrographs were imported into cryoSPARC v4^58^. Particles were picked using template picking and extracted. After multiple rounds of 2D classification, ab initio reconstruction, non-uniform refinement, 3D classification and local refinement, particle subtraction was performed to mask the micelles and flexible intracellular domain. Next, the subtracted particles were subjected to local refinement with re-centering and a new fulcrum location specified, yielding a reconstruction at 3.25 Å resolution. Finally, this portion of particles was transferred to RELION-5^60^ (https://github.com/3dem/relion/tree/ver5.0) using csparc2star.py in the UCSF pyem program packages^61^ and subjected to consensus refinement with a soft mask encompassing only the extracellular domain, yielding an extracellular domain reconstruction at 3.20 Å resolution. Validations and analyses of the density maps can be found in Supplementary Fig. S14c–g.

The resolution of the reconstruction was determined by the gold standard FSC using the FSC = 0.143 criterion in cryoSPARC v4^58^ or RELION-5^60^ (https://github.com/3dem/relion/tree/ver5.0). The 3D FSC analysis was conducted on the Remote 3DFSC Processing Server^63^ (https://3dfsc.salk.edu/). All figures depicting the maps were prepared using UCSF ChimeraX^64^ or UCSF Chimera^65^.

### Model building and refinement

The locally refined maps, sharpened by B-factors, were combined in UCSF Chimera^65^ to aid in model building. The model of the B30.2 domain of BTN2A1/3A1 in complex with HMBPP (PDB ID: 8JYE^34^), the model of BTN2A1 in complex with Vγ9Vδ2 TCR (PDB ID: 8DFW^33^), and the AlphaFold2^66^-predicted models of the BTN2A1 homodimer, BTN3A1/3A2 heterodimer and BTN3A1/3A3 heterodimer were used as initial models and rigidly fitted into corresponding density maps using USCF Chimera^65^. Several iterative rounds of real space refinement with secondary structure and geometry restraints in Phenix^67^ and manual adjustment in COOT^68^ were performed until the model fit the density well and satisfied the criteria.

To monitor overfitting, the resulting model was refined against one of the two independent half-maps (hereafter called “half map 1”) in Phenix^67^, and the refined model was then converted to a map utilizing EMAN2^69^. This map was subsequently used to compute the FSC with “half map 1” and “half map 2”. These two FSC curves are referred to as “model_vs_half_map1” and “model_vs_half_map2”, respectively. Similarly, the resulting model was refined against the summed map of the two half-maps, and the refined model was used to compute the FSC curve with the summed map, which was referred to as the “model_vs_summed map”. For each model, all three of these FSC curves were plotted. All the models in this work were validated using the Phenix.molprobity^70^ tool (Supplementary Table S1). UCSF Chimera^65^ and UCSF ChimeraX^64^ were used for structure visualization and figure preparation.

### Measurement of binding affinity

SPR was performed using a Biacore 1 K (Cytiva) to assess the binding kinetics of the WT, AAR, and AAA complexes with the G115 TCR. A CM5 chip was used to immobilize these complexes, achieving a surface density of ~5000 response units with 10 mM sodium acetate. The soluble Vγ9Vδ2 TCR complex was injected in a two-fold dilution series (27.43 μM to 0.85719 μM) at a flow rate of 30 μL/min, with a contact time of 180 s and a dissociation time of 300 s. The final response was calculated by subtracting signals from the control flow cell (BTNL3/L8) and blank injections. Data fitting was performed using a 1:1 binding kinetics model with Biacore Insight Evaluation Software (v5.0.18.22102; Cytiva), and analysis was performed using GraphPad Prism v9.

### FLIM-FRET measurement and analysis

Full-length BTN2A1, BTN3A1, and BTN3A2 were fused and separated by P2A ribosome-skipping sites (GSGATNFSLLKQAGDVEENPGP) and were subcloned into an optimized PCAG vector. Two strategies were employed to fuse fluorescent proteins. In the first strategy, CFP (moxCerulean3^71^) was fused to the amino terminus of BTN3A1, and YFP (mGold^72^) was fused to BTN2A1. The constructs included BTN2A1-*P2A*-CFP-BTN3A1-*P2A*-BTN3A2 (donor) and YFP-BTN2A1-*P2A*-CFP-BTN3A1-*P2A*-BTN3A2 (donor + acceptor). In the second strategy, CFP or YFP was fused to the carboxyl terminus of BTN3A2, generating BTN2A1-*P2A*-BTN3A1-*P2A*-BTN3A2-CFP (donor) and BTN2A1-*P2A*-BTN3A1-*P2A*-BTN3A2-YFP (acceptor).

Lenti-X 293T cells were transfected using Lipofectamine 3000 (Thermo Fisher Scientific). For the first strategy, a donor plasmid was transfected alone (donor only group), and a donor + acceptor plasmid was transfected (donor + acceptor group). In the second strategy, the donor plasmid was transfected alone (donor only group), while the donor and acceptor plasmids were co-transfected at a 1:1 DNA mass ratio (donor + acceptor group). The cells were cultured for 14 h before live-cell imaging.

Fluorescence lifetime measurements of the donor fluorophore were conducted with a Leica STELLARIS 8 microscope, which has a temporal resolution of 97 ps, a confocal scan head with FPGA electronics, 448 nm pulsed laser excitation, and fast spectral single-photon counting detectors. For FLIM-FRET analysis, FLIM images were generated by recording the average photon arrival per pixel. To ensure accuracy, a minimum of 2000 photons per pixel were collected for both the donor and acceptor acquisitions. A multi-exponential donor fit model was used to analyze time-correlated single-photon counting decay curves. The amplitude of each lifetime component was determined, and the intensity-weighted mean lifetime (τ) was calculated. For the donor-only group, CFP-expressing cells were analyzed, and the average weighted mean lifetime (τ) was measured using the n-Exponential Deconvolution model. In the donor + acceptor group, cells expressing both CFP and YFP were analyzed to determine the average lifetime within the cell membrane. FRET efficiency (*E*) was calculated using the following equation:

$$E=1-\frac{{\rm{\tau }}{\rm{AvAmp}}}{{\rm{\tau }}{\rm{D}}}$$

where τD is the lifetime of the unquenched donor and τAvAmp is the amplitude-weighted average decay time.

### AUC

AUC analysis of the purified protein samples was performed on an Optima AUC-A/I analytical ultracentrifuge (Beckman Coulter). The complex was first purified by SEC and then concentrated to a final volume of 50 μL. The samples were subsequently diluted to 380 μL in the AUC buffer (25 mM HEPES, pH 7.4, 150 mM NaCl, 0.02% GDN) and loaded onto a double-sector sapphire cell, along with 400 μL of reference buffer (25 mM HEPES, pH 7.4, 150 mM NaCl). Sedimentation velocity AUC (SV-AUC) was carried out using an An-50 Ti rotor at 40,000 rpm and 20 °C. Sedimentation profiles were monitored by measuring the absorbance at 280 nm. The data were analyzed using SEDFIT. The extinction coefficient for the 3A2 complex was calculated using the ExPASy server (https://www.expasy.org).

### Generation of stable NIH-3T3 cell lines

To generate stable NIH-3T3 cell lines expressing different BTN variants, BTN2A1, BTN3A1, and BTN3A2 were cloned and inserted into the lentiCRISPR v2 vector with a spleen focus-forming virus (SFFV) promoter. The HA tag and Flag tag were fused to the N-terminus of BTN2A1 and BTN3A1 separately, along with a mCherry tag at the C-terminus of BTN3A2, which was separated by a P2A ribosome-skipping site (ATNFSLLKQAGDVEENPGP). The lenti-plasmids, along with the pMD2. G and psPAX2, were co-transfected into Lenti-X 293T cells to pack the lentivirus. The supernatants were collected at 48 h and 72 h post-transfection. Then, the lentivirus was concentrated using 80 μg/mL protamine sulfate (Macklin) and 80 μg/mL chondroitin sulfate C (Macklin) before being transduced into NIH-3T3 cells. Transduced NIH-3T3 cells exhibiting comparable mCherry fluorescence intensities were sorted via flow cytometry. Additionally, to ensure equivalent expression levels of the WT 3A2 complex, AAR mutant, and Y127A mutant, anti-Flag (BioLegend) or anti-HA tag antibodies (BioLegend) were used.

### Multi-SIM imaging

To measure the oligomer formation and the length distribution of the 3A2 complex, NIH-3T3 cells were transduced with the pLVX-mGold-3A2 complex or the indicated mutants with a P_TRE3GS_ inducible promoter, whose activity is regulated by doxycycline. The NIH-3T3 stable cell line was prepared using the same lentivirus packaging system. For super-resolution imaging of the 3A2 complex and mutants, cells were seeded onto dishes (Cellvis) and treated with doxycycline for 18 h. The specimens were subsequently washed with PBS and fixed with 4% paraformaldehyde, followed by imaging in TIRF-SIM mode. Images were visualized and processed by Fiji ImageJ. Statistical length distribution analysis of the oligomers was performed using Prism (GraphPad).

### Human Vγ9Vδ2 T-cell expansion

The study involving human biological materials was performed in accordance with relevant ethical guidelines and regulations. Cryopreserved human PBMCs were purchased from Shanghai Sailybio Medical Co., Ltd., as approved by the Shanghai Liquan Hospital Institutional Review Board (project no. XF-WBC-220809). PBMCs (1 × 10^6^) were cultured in 12-well plates with 5 μM zoledronate treatment (Sigma). The medium was replaced with fresh medium (OptiVitro® T cell medium; Excell Bio) supplemented with human IL-2 and IL-15 (PeproTech) every 3 days. Vγ9Vδ2 T cells (purity > 90%) were expanded at 15 days and confirmed by anti-human CD3 (BD Pharmingen) and anti-human TCR Vδ2-FITC (BioLegend) antibodies. The protocol was approved by the Institutional Review Board of Westlake University (project no. 20241010xwz).

### Generation of a K562 *BTN3A1*^*–/–*^ knock-out cell line

K562 *BTN3A1* knockout cells were generated by CRISPR–Cas9-mediated genome editing. Briefly, 2 × 10^4^ K562 cells were transfected with 0.25 μg of SpyCas9 mRNA and 0.25 μg of sgRNA (target sequence: AGAGACCAUGGAGCUGAAGU) using ProteanFect Max (Nanoportal Biotech) following the manufacturer’s instructions. Three days after transfection, single cells were sorted into 96-well plates using a BD FACSAria III cell sorter. Clonal populations were expanded for two weeks and screened by PCR and Sanger sequencing. A *BTN3A1*^–/–^ clone was identified and used for subsequent experiments.

### Functional assessment of human polyclonal Vγ9Vδ2 T-cell activation

NIH-3T3 and K562 cells expressing the SFFV-3A2 complex or the indicated mutants were seeded in 24-well plates for 12 h, followed by co-incubation with in vitro-expanded Vγ9Vδ2 T cells from PBMCs (5 × 10^5^) ± zoledronate (5 μM). After 24 h, culture supernatants were collected from Vγ9Vδ2 T cells. The IFN-γ expression levels in the medium were measured using a human IFN-γ ELISA kit (Beyotime), and the absorbance was measured at 450 nm by a multimode microplate reader (Varioskan LUX, Thermo Fisher Scientific). Vγ9Vδ2 T cells were washed with FACS buffer and stained with anti-human TCR Vδ2-FITC and anti-human CD25-APC for 1 h (BD Pharmingen). Flow cytometry analysis of CD25 expression was conducted on a Cyto-FLEX LX-5L2 (Beckman). The raw data were analyzed using FlowJo (BD Biosciences).

### Vγ9Vδ2 TCR tetramer preparation and tetramer staining

The soluble Vγ9Vδ2 TCR with an Avi tag was enzymatically biotinylated using the biotin ligase BirA (made in house). The biotinylated Vγ9Vδ2 TCR was then incubated with streptavidin–FITC (Solarbio) at a 4:1 molar ratio at 4 °C overnight. NIH-3T3 cells expressing the WT 3A2 complex, Y127A mutant or AAR mutant were plated in 24-well plates and treated with or without zoledronate for 24 h. For Vγ9Vδ2 TCR tetramer staining, the cells were digested with 0.25% trypsin-EDTA, washed three times with FACS buffer, and subsequently incubated with 5 μg of Vγ9Vδ2 TCR-streptavidin complex at 4 °C for 1 h. Data acquisition was performed using a Cyto-FLEX LX-5L2 flow cytometer (Beckman).

### Assessment of BTN3A surface expression among primary cell lines

The surface expression of BTN3A was evaluated in a panel of cell lines comprising HEK293T, K562, HeLa, and RPMI-8226 cells, as well as BTN3A-transduced NIH-3T3 cells and PBMCs. Briefly, 1 × 10^5^ live cells were incubated with a rabbit monoclonal anti-BTN3A antibody (Abcam, ab319101; clone EPR28070-58), which cross-reacted with BTN3A1, BTN3A2, and BTN3A3 isoforms, at a 1:50 dilution for 2 h on ice. The cells were subsequently washed twice with PBS and stained with an ABflo® 647-conjugated goat anti-rabbit IgG (H + L) secondary antibody (ABclonal, AS010) at a 1:2000 dilution for 1 h on ice. Fluorescence histograms were acquired using a CytoFLEX LX-5L flow cytometer (Beckman Coulter) and analyzed using FlowJo software.

### In situ Duolink® PLAs

Duolink® PLAs were performed on K562 cell lines following the manufacturer’s standard protocol. Briefly, 5 × 10^5^ cells that were either left untreated or pre-treated with pAg (10 μM zoledronate or 5 μM HMBPP) were harvested, fixed with 4% paraformaldehyde (PFA; Beyotime, P0098) for 15 min, and permeabilized using an immunostaining permeabilization solution (Beyotime, P0097). The cells were then blocked for 1 h on ice with a combination of Duolink® Blocking Solution and human Fc receptor blocking reagent (Beyotime, C1752S). Without intermediate washing, the cells were incubated with a rabbit monoclonal anti-BTN3A antibody (Abcam, ab319101; clone EPR28070-58), which cross-reacted with BTN3A1, BTN3A2, and BTN3A3 isoforms, followed by Duolink® PLA PLUS (Sigma-Aldrich, DUO92002) and MINUS (Sigma-Aldrich, DUO92005) oligonucleotide-conjugated donkey anti-rabbit secondary antibodies. Upon binding to proximal epitopes, the PLUS and MINUS oligonucleotides hybridize and undergo ligation to form a circular DNA template. This circle then serves as a substrate for rolling circle amplification, generating a tethered DNA concatemer that is subsequently hybridized with fluorescent detection oligonucleotides. The resulting discrete fluorescent spots, which are indicative of individual PLA events, were detected using the Duolink® FlowPLA Detection Kit (Orange; Sigma-Aldrich, DUO94003) with 561 nm excitation on a CytoFLEX LX-5L2 flow cytometer (Beckman Coulter). NIH-3T3 cells subjected to identical treatment conditions served as the negative control. To minimize technical variation across independent experimental batches, we performed centering normalization on the data. Briefly, for each treatment group, the values in each batch were first subtracted from the mean of that batch and then added to the mean of all batch means within the group. The data were analyzed using GraphPad Prism 9, and the results are expressed as the geometric mean fluorescence intensity (gMFI) for the pAg-treated group vs the untreated group.

## Supplementary information

Source Data

Supplementary Information

Supplementary Video 1

## Data Availability

All cryo-EM maps have been deposited in the Electron Microscopy Data Bank (EMDB; https://www.ebi.ac.uk/pdbe/emdb/), and all refined atomic models have been deposited in the Protein Data Bank (PDB; https://www.rcsb.org/). Accession numbers for all EMDB and PDB depositions are listed in Supplementary Table S1. All materials are available from the corresponding authors upon reasonable request.
