# Supplementary material for: Phosphoantigen-driven dissociation of butyrophilin oligomers activates γδ T cells
Source: Cell Discov. 2026 Aug 11;12:59. doi: 10.1038/s41421-026-00911-7 (PMC13457854; doi:10.1038/s41421-026-00911-7)
Supplement: Supplementary file 1 [file 41421_2026_911_MOESM2_ESM.pdf]

Supplementary information for
Phosphoantigen-Driven Dissociation of Butyrophilin Oligomers Activates γδ T Cells

Weizhi Xin[1,2,3,4*], Bangdong Huang[1,2,3,4*], Weijie Gao[2,3,4], Wenjia Zhang[2,3,4], Yundi Hu[2,3,4], Yuehua Liu[2,3,4], Enyuan Liang[5], Jin Chen[5], Yigong Shi[2,3,4,6], Qiang Su[5]†, Qiang Zhou[2,3,4]†

[1]Fudan University, Shanghai, China.

[2]Research Center for Industries of the Future, Center for Infectious Disease Research, Zhejiang Key Laboratory of Structural Biology, State Key Laboratory of Gene Expression, School of Life Sciences, Westlake University, Hangzhou, Zhejiang, China.

[3]Westlake Laboratory of Life Sciences and Biomedicine, Hangzhou, Zhejiang, China.

[4]Institute of Biology, Westlake Institute for Advanced Study, Hangzhou, Zhejiang, China.

[5]Institute of Bio-Architecture and Bio-Interactions (IBABI), Shenzhen Medical Academy of Research and Translation (SMART), Shenzhen, Guangdong 518107, China.

[6]Beijing Advanced Innovation Center for Structural Biology, Tsinghua-Peking Joint Center for Life Sciences, School of Life Sciences, School of Medicine, Tsinghua University, Beijing, China.

*These authors contributed equally: Weizhi Xin, Bangdong Huang.

†Corresponding author. Email: suqiang@smart.org.cn (Q.S.); zhouqiang@westlake.edu.cn (Q.Z.)

This file includes:

Supplementary Fig. S1-17

Supplementary Table S1

Supplementary Video S1

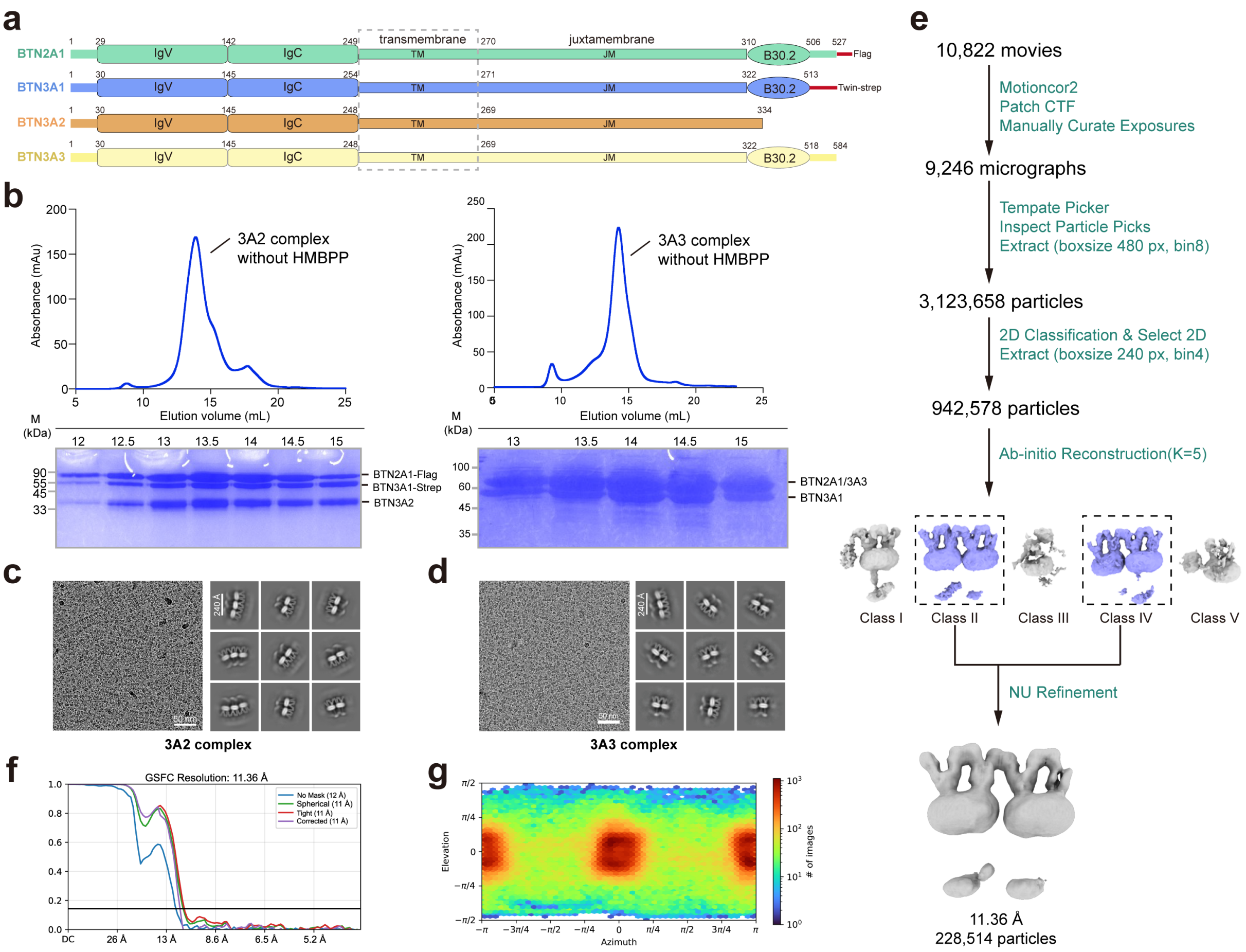

Supplementary Fig. S1 Biochemical characterization of BTN complex. a, Schematic representation of domain organization of BTN2A1, BTN3A1, BTN3A2, and BTN3A3. TM: transmembrane; JM: juxtamembrane. Domain boundaries are indicated. **b,** Representative size exclusion chromatography (SEC) profile of the 3A2 complex or 3A3 complex (left and right, top panel) and analysis of the relevant fractions by Coomassie blue staining (left and right, bottom panel) in the absence of HMBPP. **c,d,** Representative cryo-EM image and 2D class averages of the 3A2 complex (**c**) or the 3A3 complex (**d**). **e,** Summary of the cryo-EM data processing workflow for the 3A2 complex oligomer from (**b**). **f,** FSC curves of the overall reconstruction of the 3A2 complex oligomer. **g,** Angular distribution of particles used for the final reconstruction.

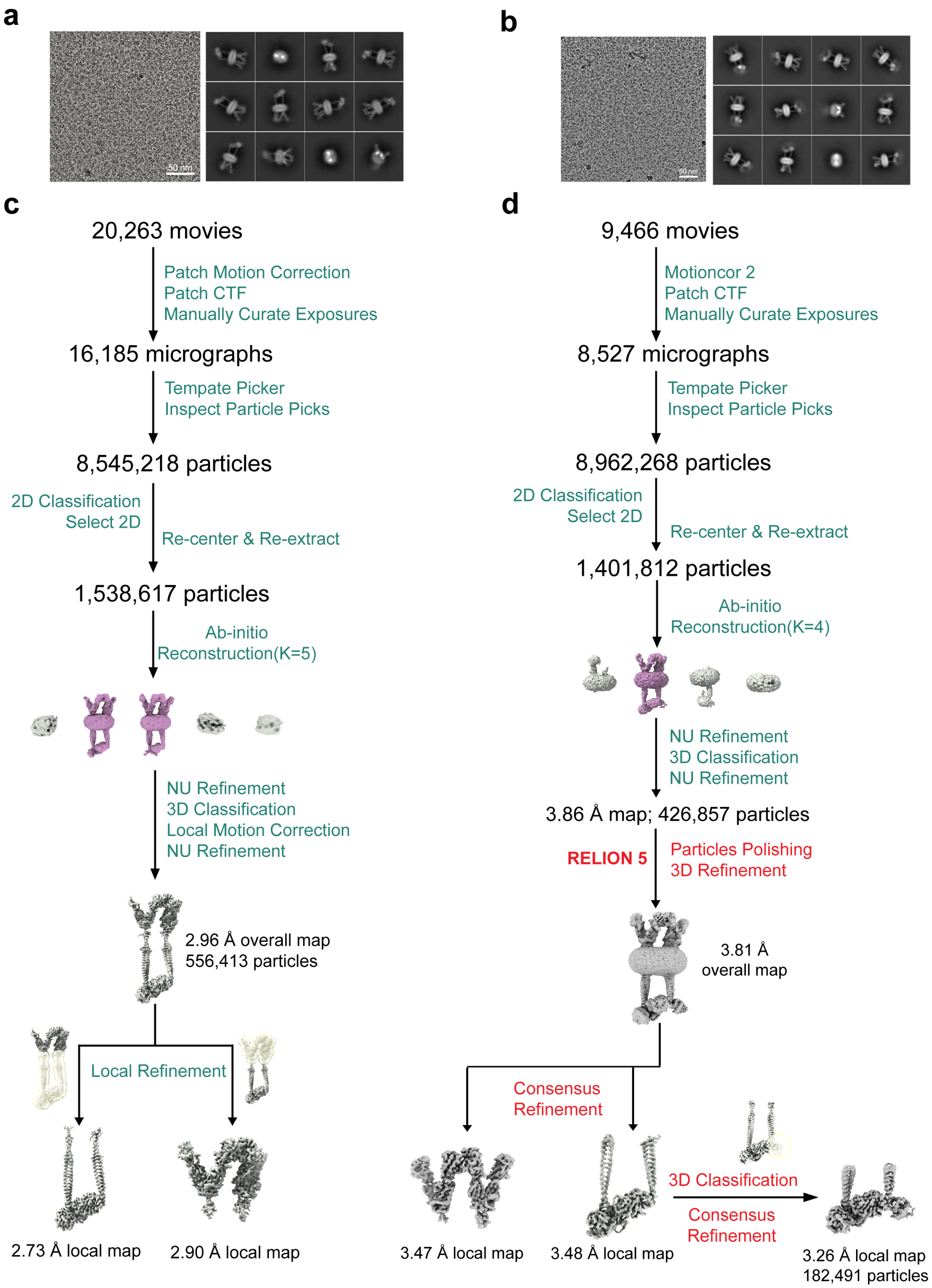

Supplementary Fig. S2 Cryo-EM analysis of pAg-bound BTN complexes. a,b, Representative cryo-EM images and 2D class averages of the 3A2 complex (**a**) and the 3A3

complex (**b**) in the presence of 4 μM HMBPP. **c,d,** Workflow of the cryo-EM data processing for the 3A2 complex (**c**) and the 3A3 complex (**d**) in the presence of HMBPP. Red annotations denote data processing steps conducted in RELION, whereas green annotations indicate computational procedures implemented using cryoSPARC.

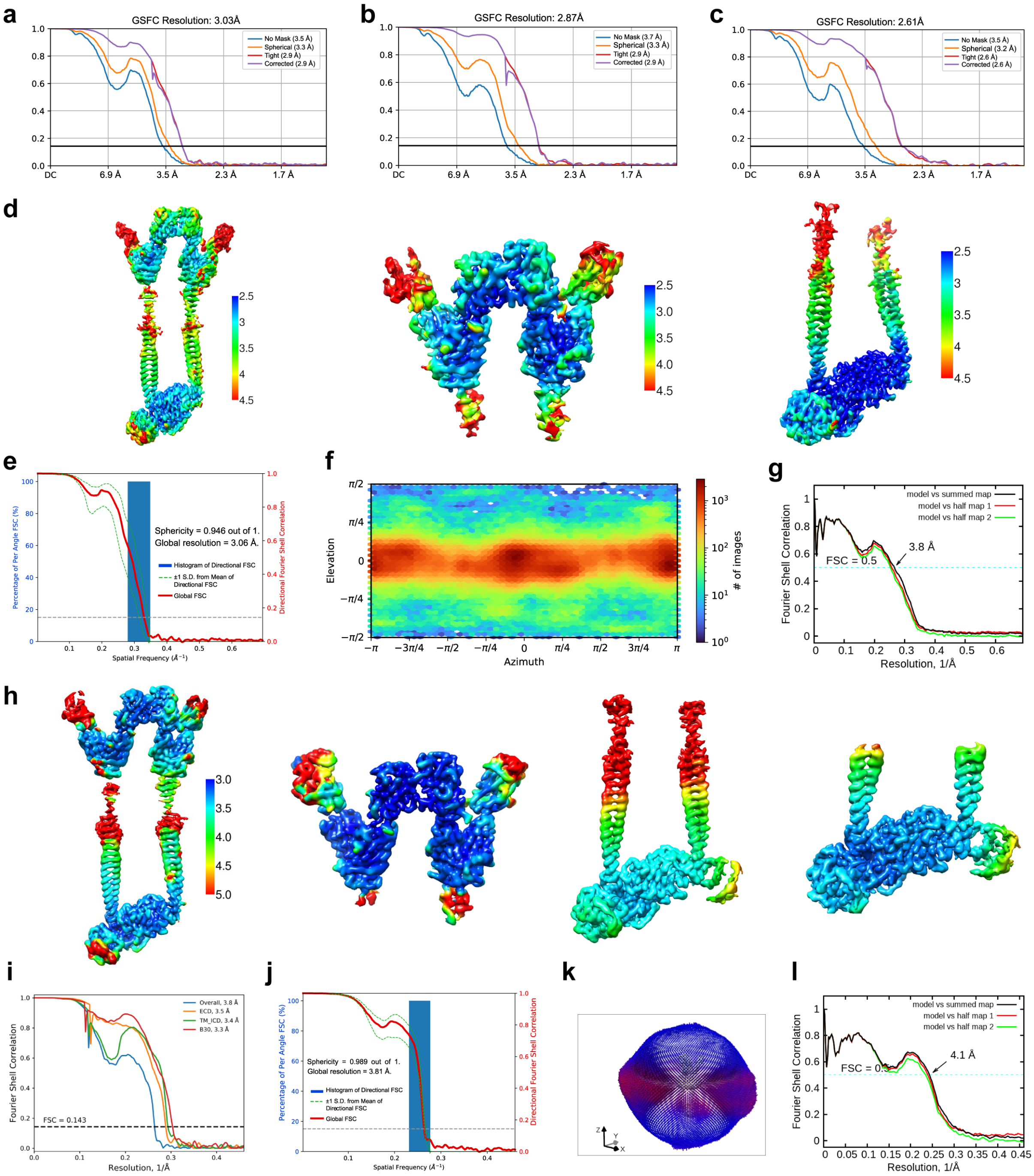

Supplementary Fig. S3 Validation of cryo-EM maps and atomic models of pAg-bound BTN complexes. a–c, Fourier shell correlation (FSC) curves for the overall refinement (**a**), extracellular domain (ECD) local refinement (**b**), and intracellular domain (ICD) local refinement (**c**) of the 3A2 complex. **d,** Local resolution estimation of the 3A2 complex overall map (left), ECD-focused map (middle), and ICD-focused map (right). Noise was manually removed for clarity. **e,** The 3DFSC sphericity plot of the 3A2 complex overall map generated

by 3DFSC. **f,** Angular distribution of particles for the final reconstruction of the overall 3A2 complex. **g,** Model versus map FSC curves for the 3A2 complex. **h,** Local resolution estimation of the 3A3 complex overall map (left), ECD-focused map (middle), ICD-focused map (right), and B30.2-focused map (far right). Noise was manually removed for clarity. **i,** FSC curves for overall refinement and consensus refinements of the 3A3 complex. Dashed line indicates FSC = 0.143. **j,** The 3DFSC sphericity plot of 3A3 complex overall map generated by 3DFSC. **k,** Angular distribution of particles for the final reconstruction of the overall 3A3 complex. **l,** Model versus map FSC curves for the 3A3 complex. See Methods for specific details regarding FSC curve computation in (**g**) and (**i**).

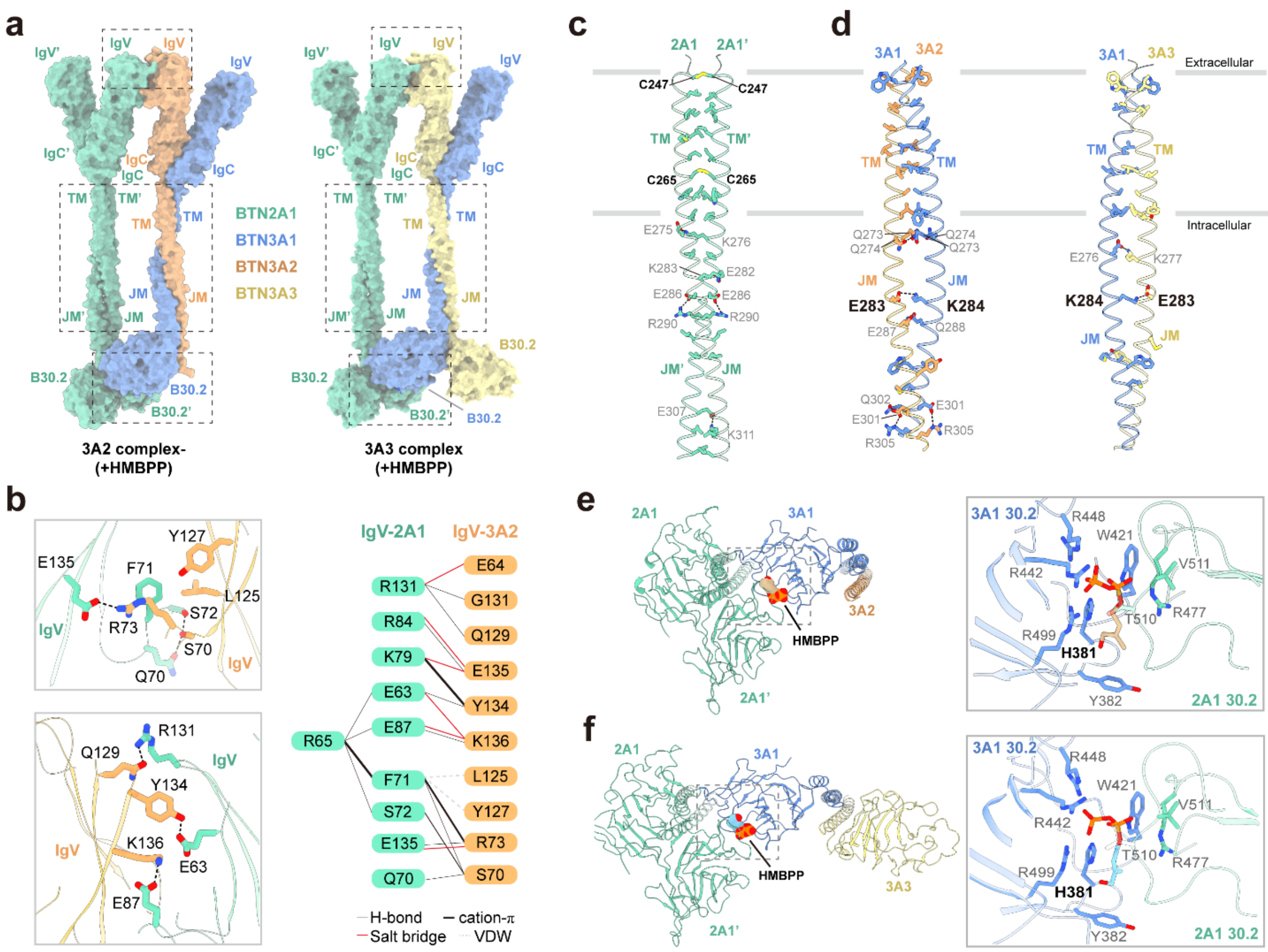

Supplementary Fig. S4 Interchain interactions determine BTN complex assembly. a, Surface representation of the pAg-bound 3A2 complex (left) or the pAg-bound 3A3 complex (right). Dashed boxes highlight critical regions for BTN complex assembly. Subunit rendering: BTN2A1 (medium aquamarine), BTN3A1 (cornflower blue), BTN3A2 (sandy brown), and BTN3A3 (khaki). This color scheme is consistent throughout the work. **b,** Close-up views of the IgV domain interface between BTN2A1 and BTN3A2 (left). Residues are shown as sticks, with hydrogen bonds, salt bridges, and Cation-π interactions indicated by dashed lines. The intricate interaction network between IgV_{BTN2A1} and IgV_{BTN3A2} is shown through a connection diagram of related residues (right). **c,d,** Close-up views of the interface interactions between the TM/JM domains of the BTN2A1 homodimer (**c**), BTN3A1/3A2 heterodimer (**d**, left), and BTN3A1/3A3 heterodimer (**d**, right). Models are shown in cartoon style, with interacting residues depicted as sticks. Hydrogen bonds are dashed, and disulfide bonds are highlighted in yellow. TM: transmembrane; JM: juxtamembrane. **e,f,** HMBPP binds to the B30.2 domain of

BTN2A1 and BTN3A1 B30.2 domains within the 3A2 complex (**e**, left) or the 3A3 complex (**f**, left). The HMBPP is shown in sphere style. Close-up views of the HMBPP binding pocket within the BTN2A1 and BTN3A1 domains (**e** and **f**, right).

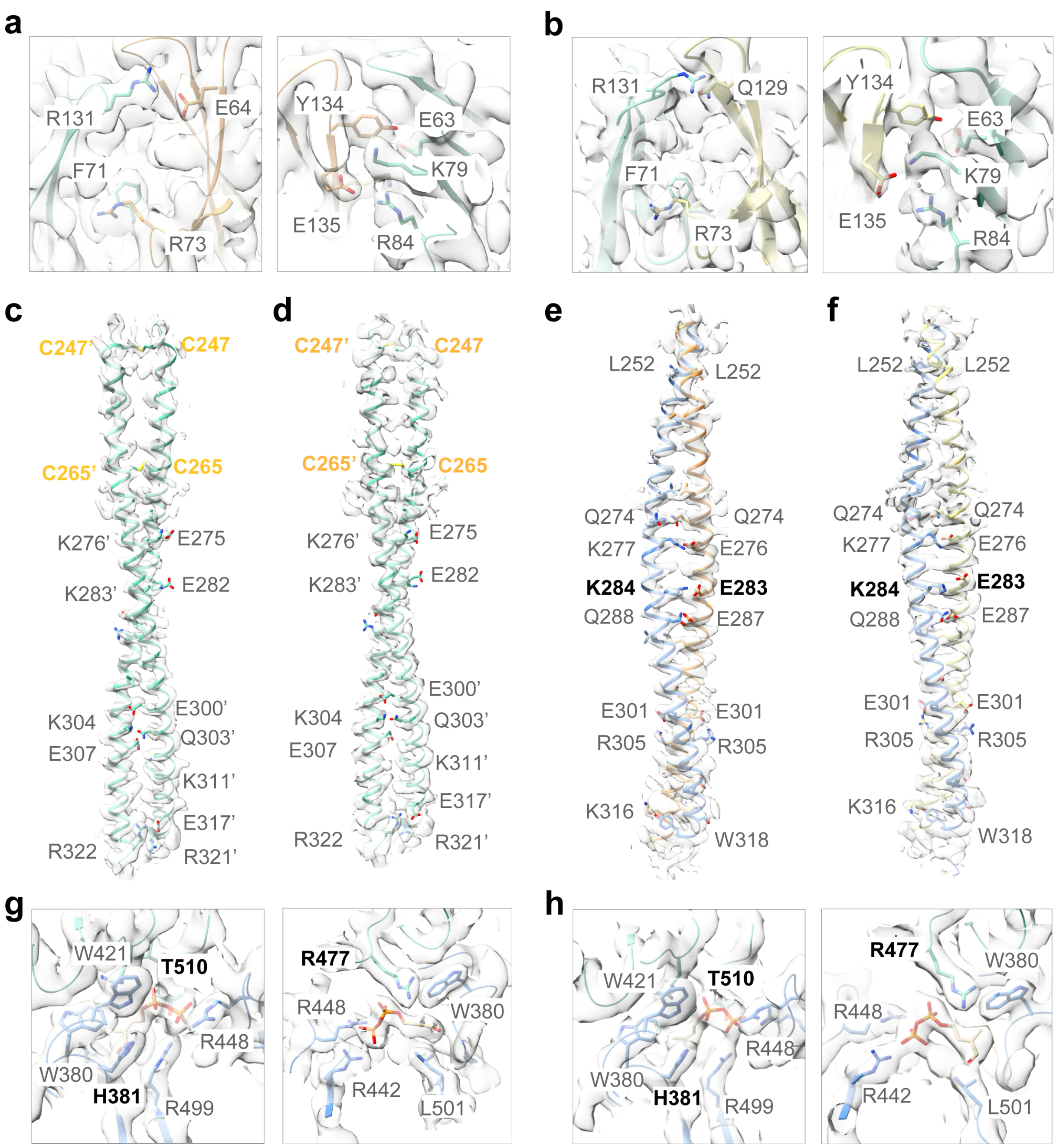

Supplementary Fig. S5 Representative cryo-EM maps of pAg-bound BTN complexes. a, Close-up view of the interface between IgV$_{BTN2A1}$ and IgV$_{BTN3A2}$, shown in two views, with the map contoured at 8 σ. **b,** Close-up view of the interface between IgV$_{BTN2A1}$ and IgV$_{BTN3A3}$, shown in two views, with the map contoured at 8 σ. **c,d,** Close-up views of the interface between the TM/JM$_{BTN2A1}$ and TM/JM$_{BTN2A1'}$ regions in the 3A2 complex (**c**) and 3A3 complex (**d**), with the maps contoured at 6 σ. **e,f,** Close-up views of the interface between the TM/JM$_{BTN3A1}$ and TM/JM$_{BTN3A2}$ or TM/JM$_{BTN3A3}$ regions in the 3A2 complex (**e**) and 3A3

complex (**f**), with the maps contoured at 8 σ. **g,h,** Close-up views of the HMBPP binding pockets in the 3A2 complex (**g**) and 3A3 complex (**h**), shown in two views, with the maps contoured at 11 σ. Side chains of residues involved in bridging interactions are labeled and shown as sticks.

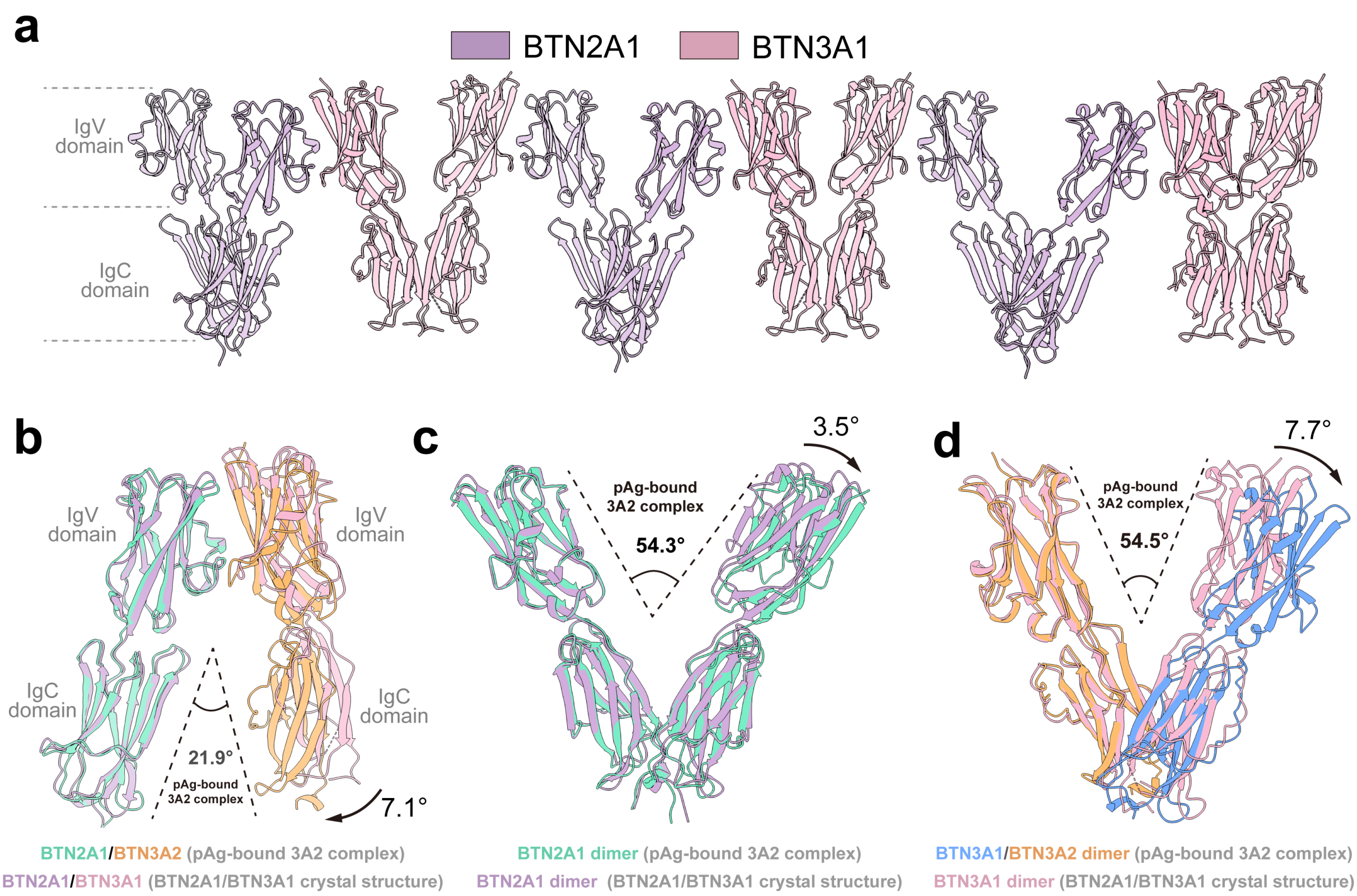

Supplementary Fig. S6 Structural comparison of the extracellular regions between the pAg-bound 3A2 complex and the BTN2A1/BTN3A1 complex. a, The high-order oligomer (PDB ID: 8DFX) in the crystal structure of BTN2A1 and BTN3A1 extracellular domain complex. **b,** Comparison of the interchain angle between the Ig-like domains of BTN2A1 and BTN3A1. In the pAg-bound 3A2 complex, the BTN2A1/BTN3A1 angle is decreased by 7.1° compared to that in the crystal structure of BTN2A1 and BTN3A1 extracellular domain complex. **c,** Comparison of the dimerization angle in the extracellular region of the BTN2A1 dimer. The BTN2A1 dimer angle in the pAg-bound 3A2 complex is increased by 3.5° relative to that of the BTN2A1 dimer in the crystal structure of BTN2A1 and BTN3A1 extracellular domain complex. **d,** Comparison of the dimerization angle in the extracellular region of the BTN3As dimer. The BTN3A1/BTN3A2 angle in the pAg-bound 3A2 complex is increased by 7.7° compared to that of the BTN3A1 dimer in the crystal structure of BTN2A1 and BTN3A1 extracellular domain complex.

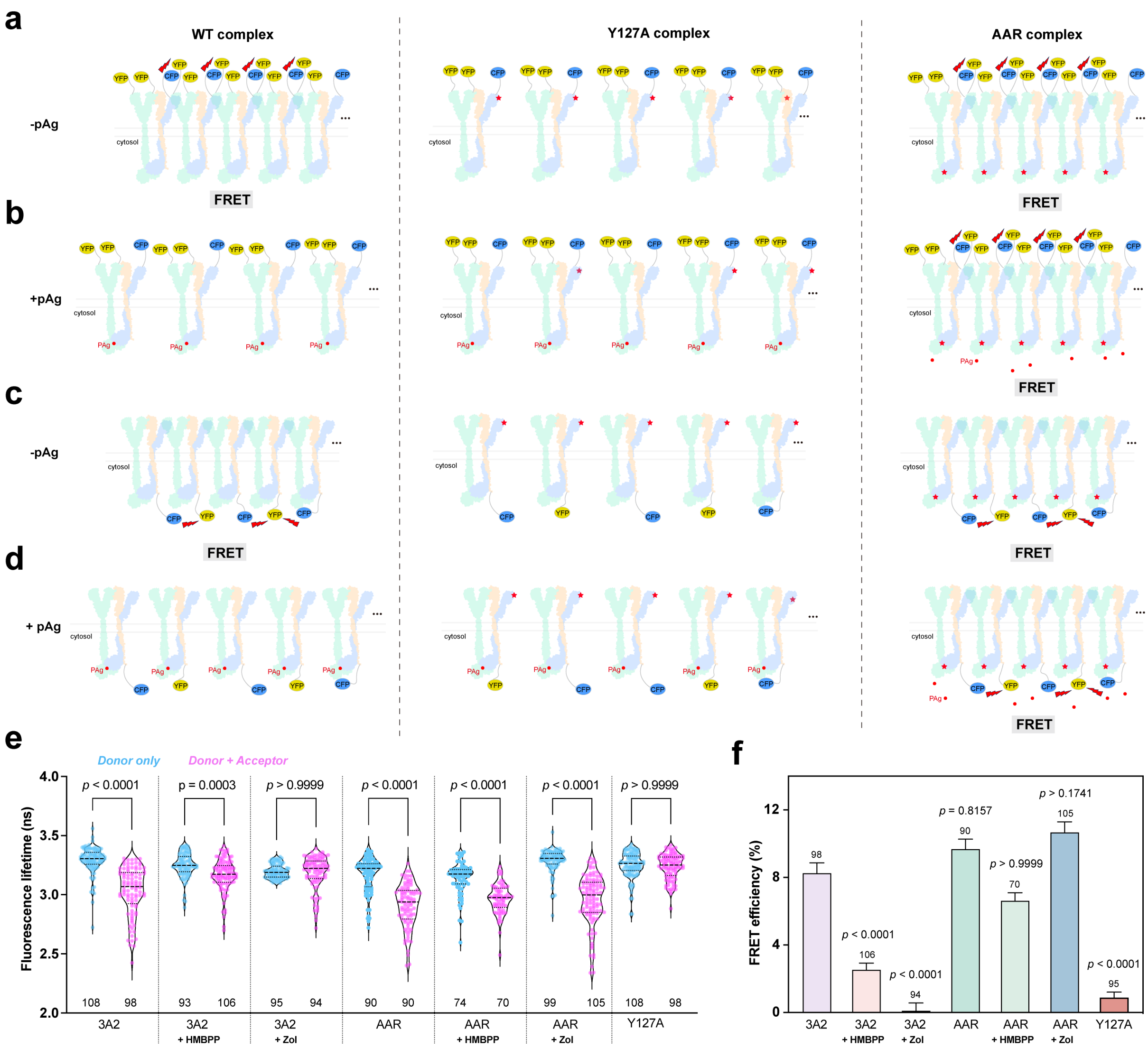

Supplementary Fig. S7 Design of FLIM-FRET assays to evaluate the oligomerization state of the 3A2 complex on cell membrane. a,b, CFP and YFP are fused to the N termini of BTN3A1 and BTN2A1, respectively. In the absence of pAg, FRET occurs in both the WT and AAR complexes (**a**), while FRET is diminished in the WT complex but still occurs in the AAR complex in the presence of pAg (**b**). **c,d,** CFP and YFP are fused to the C termini of BTN3A2. FRET occurs in both the WT and AAR complexes in the absence of pAg (**c**). In contrast, FRET is diminished in the WT complex but still occurs in the AAR complex (**d**). The mutations are indicated by red pentagrams. **e,** Fluorescence lifetime of C-terminal CFP was measured in Lenti-X 293T cells using FLIM-FRET. “Donor only” refers to cells transfected with C-terminal CFP-labeled BTN3A2. “Donor + Acceptor” refers to cells co-expressing both C-terminal CFP-

labeled and C-terminal YFP-labeled BTN3A2. The N value is provided for each group (mean ± SEM). **f,** FRET efficiency was determined in cells expressing WT 3A2 complex and its variant, using the same constructs as in (**e**). The mean fluorescence lifetime in each "Donor only" group was used to calculate FRET efficiency in each "Donor + Acceptor" group. The N value is provided for each group (mean ± SEM). *P* values were calculated using the Kruskal-Wallis test with Dunn's multiple comparisons post-test in (**e** and **f**). Y127A, the 3A2 complex variant harboring a BTN3A1-Y127A mutation to disrupt the oligomeric assembly; AAR, the 3A2 complex variant harboring BTN3A1-H381R/BTN2A1-R477A/T510A mutations to abolish HMBPP binding. Zol, zoledronate.

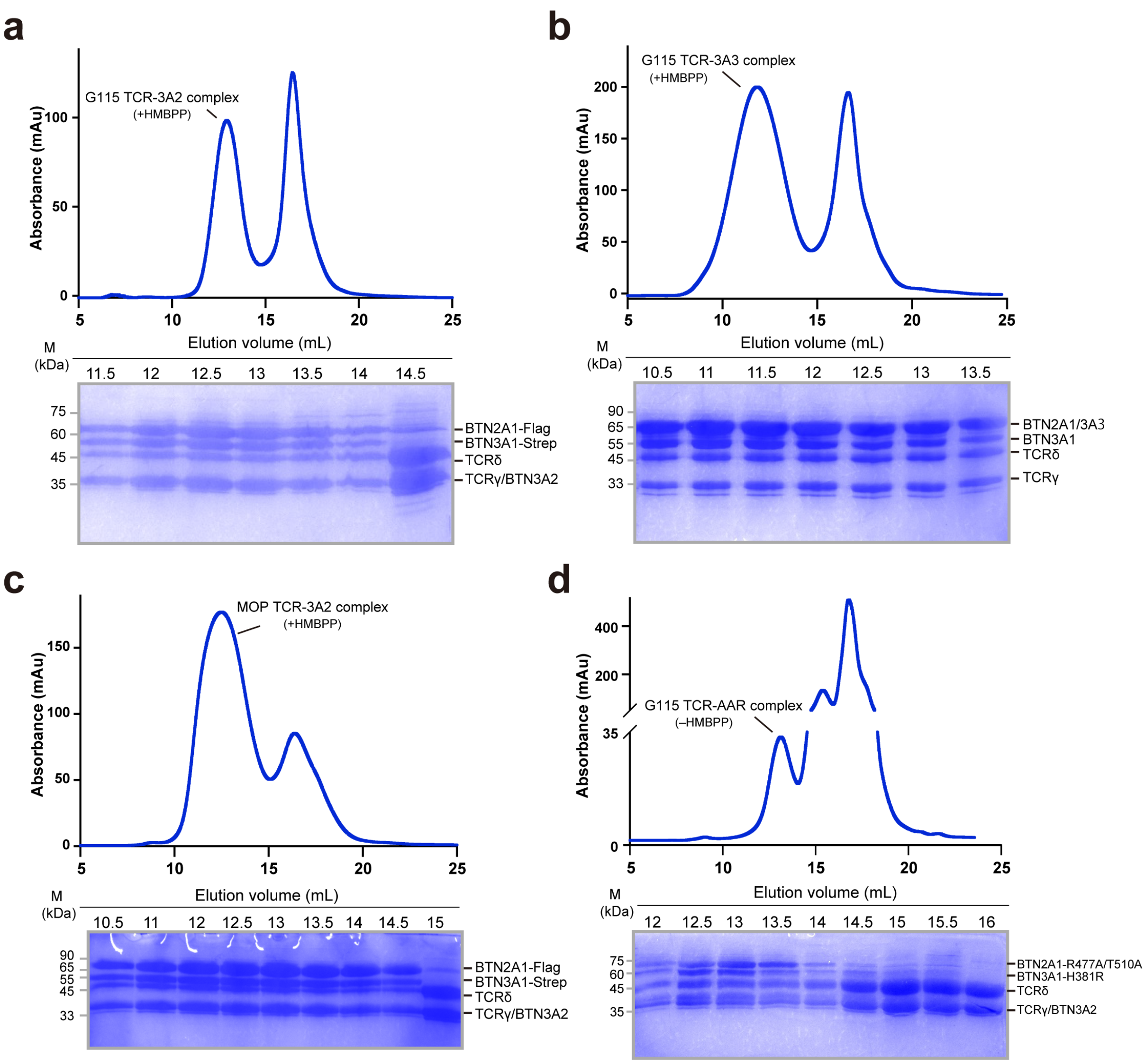

Supplementary Fig. S8 Biochemical characterization of the pAg-bound TCR–BTN complex and the TCR–AAR complex. a–d, Representative size exclusion chromatography (SEC) profiles (top panels) and Coomassie blue staining analyses of corresponding fractions (bottom panels) for the pAg-bound G115 TCR–3A2 complex (**a**), the pAg-bound G115 TCR–3A3 complex (**b**), the pAg-bound MOP TCR–3A2 complex (**c**), and the G115 TCR–AAR complex (**d**). AAR denotes the 3A2 complex variant containing BTN3A1-H381R, BTN2A1-R477A, and T510A mutations to eliminate HMBPP binding. HMBPP was added at a final concentration of 4 µM in (**a–c**). See Methods for details on protein purification.

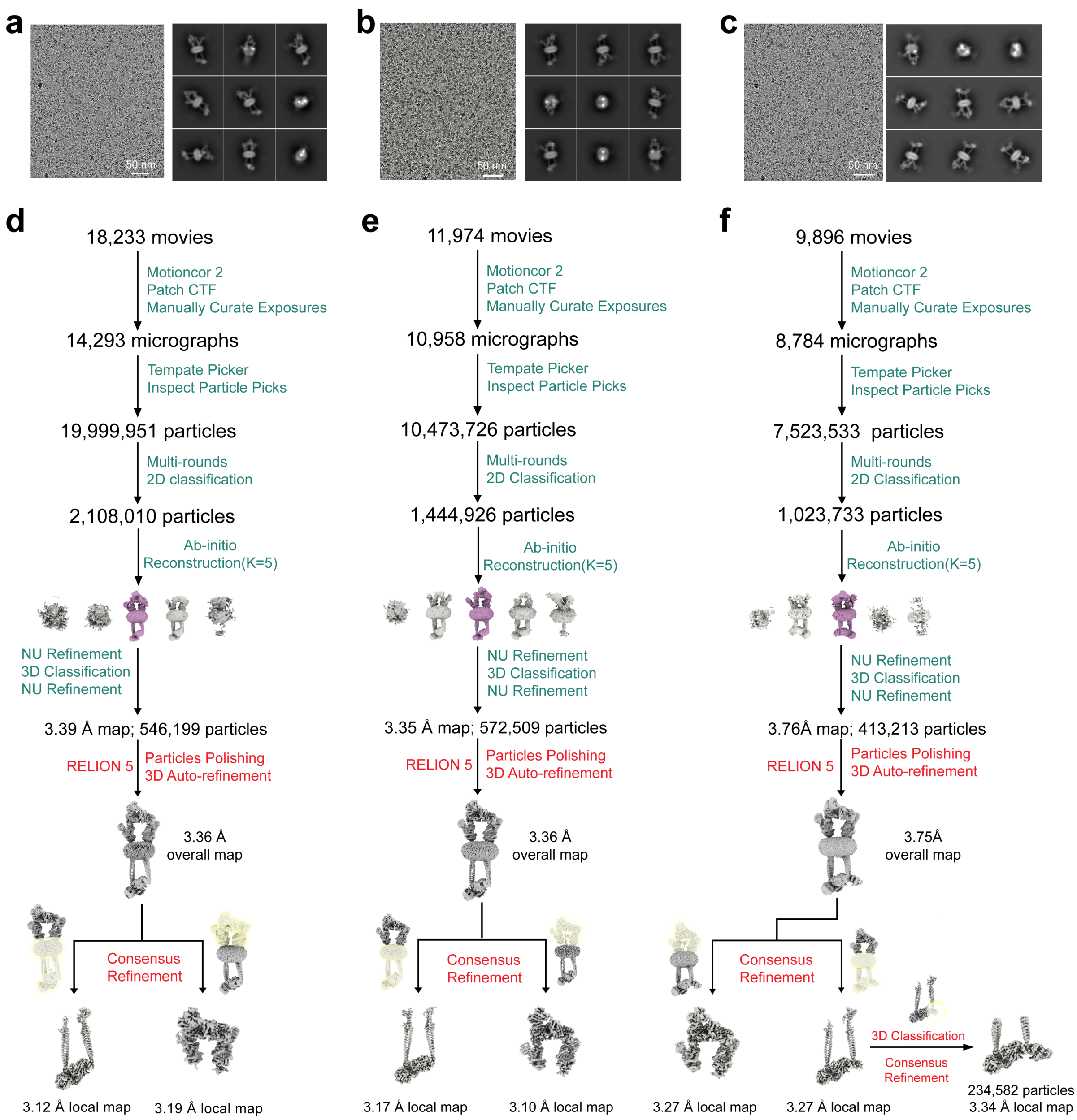

Supplementary Fig. S9 Cryo-EM analysis of pAg-bound Vγ9Vδ2 TCR–BTN complexes. **a–c,** Representative cryo-EM images and 2D class averages of the pAg-bound G115 TCR–3A2 complex (**a**), pAg-bound MOP TCR–3A2 complex (**b**), and pAg-bound G115 TCR–3A3 complex (**c**). **d–f,** Workflow of the cryo-EM data processing for the pAg-bound G115 TCR–3A2 complex (**d**), the pAg-bound MOP TCR–3A2 complex (**e**), and the pAg-bound G115 TCR–3A3 complex (**f**).

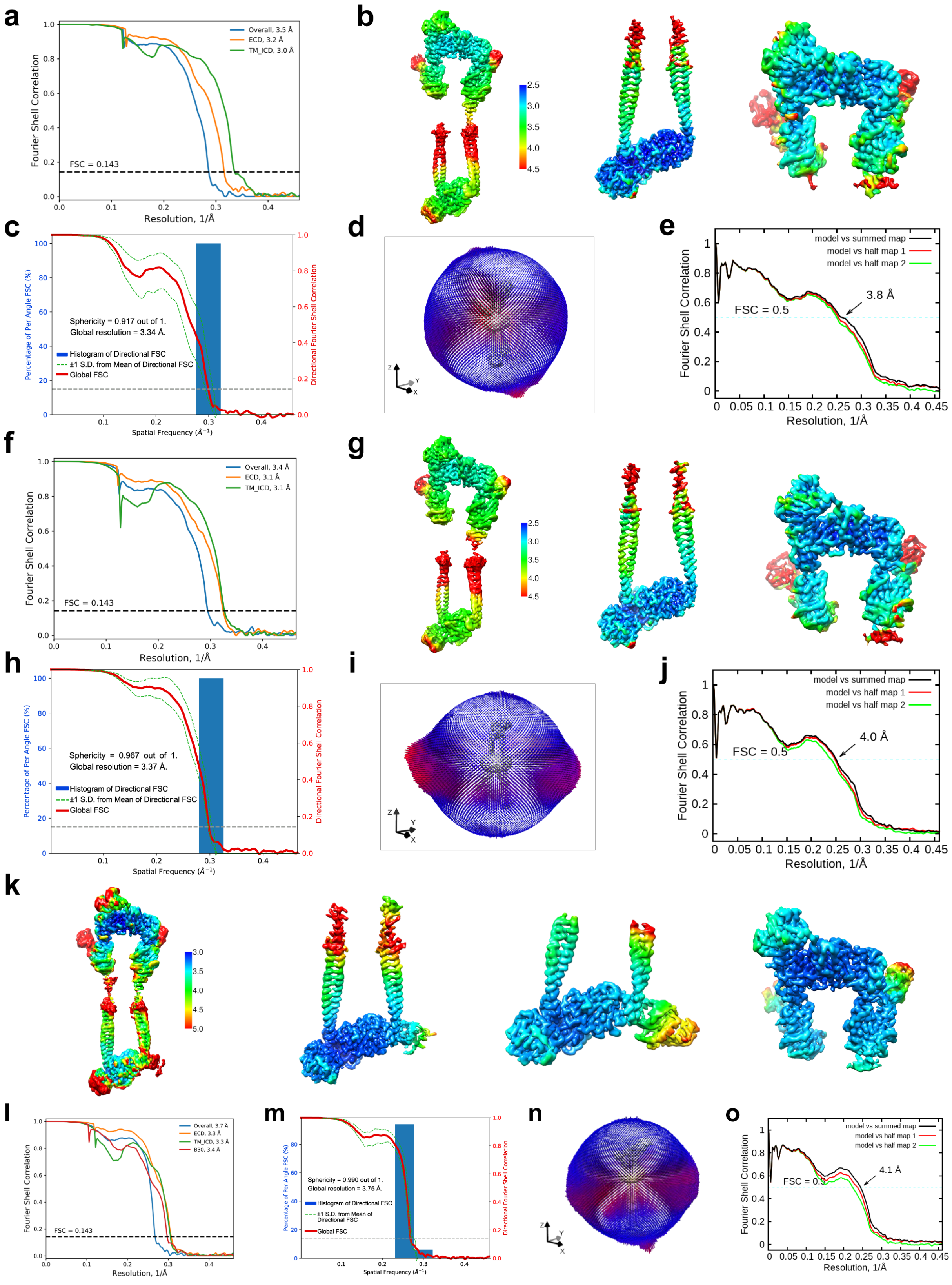

Supplementary Fig. S10 Validation of cryo-EM maps and models of pAg-bound Vγ9Vδ2 TCR–BTN complexes. a, FSC curves of overall refinement and consensus refinements for the pAg-bound G115 TCR–3A2 complex. **b,** Local resolution estimation for the pAg-bound G115

TCR–3A2 complex overall map (left), ICD-focused map (middle), and ECD-focused map (right). Noise was manually removed for clarity. **c,** 3DFSC sphericity plot generated by 3DFSC for the overall pAg-bound G115 TCR–3A2 complex. **d,** Angular distribution of particles for the final reconstruction. **e,** Model versus map FSC curves of the overall pAg-bound G115 TCR–3A2 complex. **f,** FSC curves of overall refinement and consensus refinements for the pAg-bound MOP TCR–3A2 complex. **g,** Local resolution estimation for the MOP TCR–3A2 complex overall map (left), ICD-focused map (middle), and ECD-focused map (right). Noise was manually removed for clarity. **h,** 3DFSC sphericity plot generated by 3DFSC for the overall pAg-bound MOP TCR–3A2 complex. **i,** Angular distribution of particles for the final reconstruction. **j,** Model versus map FSC curves for the MOP TCR–3A2 complex. **k,** FSC curves of overall refinement and consensus refinements for the pAg-bound G115 TCR–3A3 complex. **l,** Local resolution estimation for the G115 TCR–3A3 complex overall map (left), ICD-focused map (middle), and ECD-focused map (right). Noise was manually removed for clarity. **m,** 3DFSC sphericity plot generated by 3DFSC for the overall pAg-bound G115 TCR–3A3 complex. **n,** Angular distribution of particles for the final reconstruction. **o,** Model versus map FSC curves for the MOP TCR–3A3 complex.

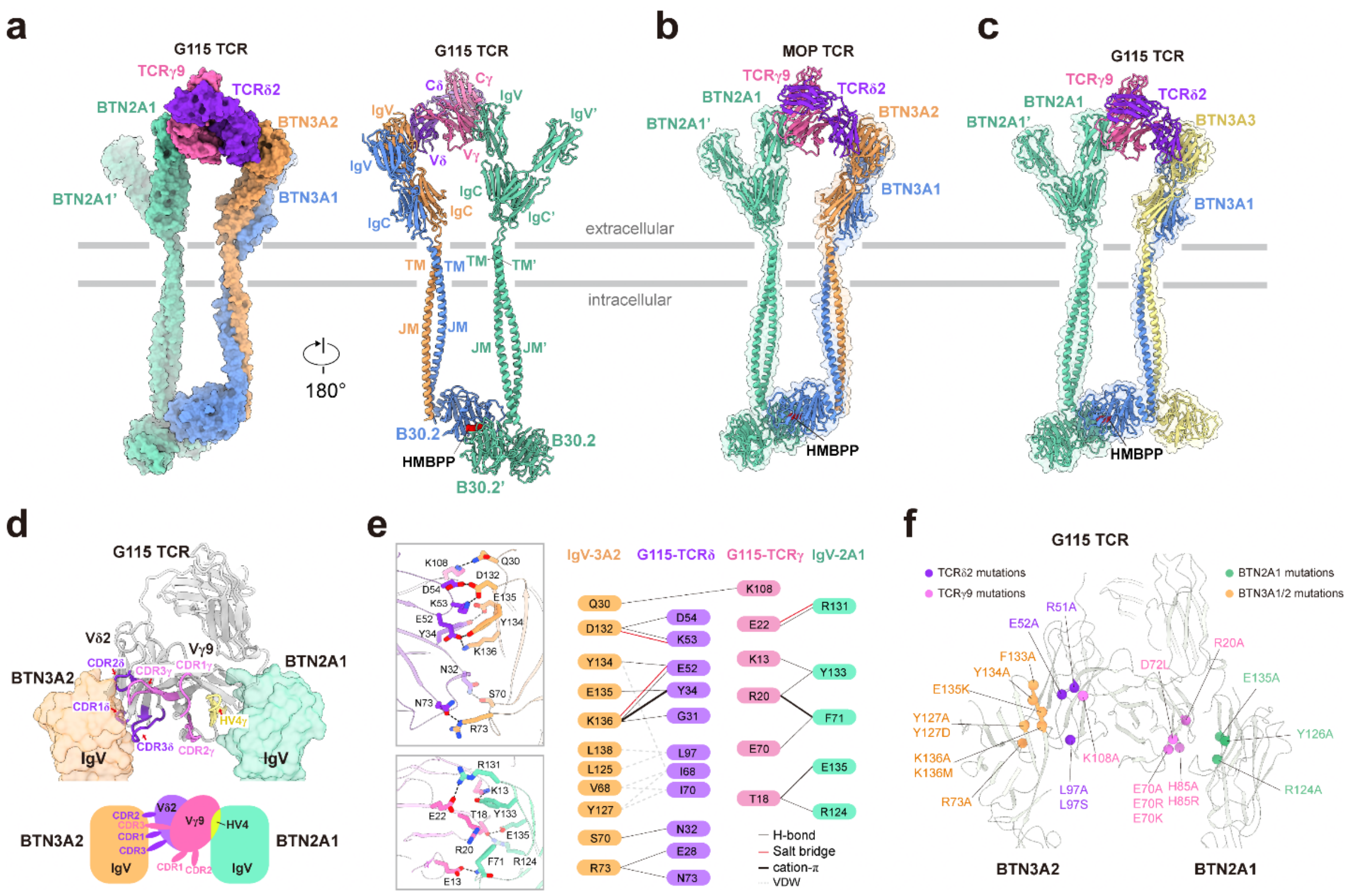

Supplementary Fig. S11 Vγ9Vδ2 TCR binds to the ectodomain of the BTN complex involving CDR loops and HV4 region. a, Overall structure of Vγ9Vδ2 TCR (G115 prototype) in complex with the pAg-bound 3A2 complex. The variable domains of the TCR are intricately positioned at the IgV_{BTN2A1}–IgV_{BTN3A2} interface. The structure is shown as a surface (left) or cartoons (right), with HMBPP depicted as a red sphere. **b,** Overall structure of Vγ9Vδ2 TCR (MOP prototype) in complex with the pAg-bound 3A2 complex. BTN molecules are shown as cartoons in the transparent surface, with TCR presented as cartoons and HMBPP shown as red sphere. **c,** Overall structure of Vγ9Vδ2 TCR (G115 prototype) in complex with the pAg-bound 3A3 complex. Subunit colors are as follows: BTN2A1 (medium aquamarine), BTN3A1 (cornflower blue), BTN3A2 (sandy brown), BTN3A3 (khaki), TCRδ (blue violet), TCRγ (hot pink). **d,** The CDR1, CDR2, CDR3, and HV4 regions of the G115 TCR are involved in recognizing the 3A2 complex. The CDRδ, CDRγ, and HV4γ regions are colored blue violet, hot pink, and yellow, respectively. **e,** Close-up views of the interfaces between the 3A2

complex and G115 TCR (left two panels). The intricate interaction network is depicted by a connection diagram of related residues (right panel). **f,** Mapping of mutations onto the G115 TCR–3A2 complex structure.

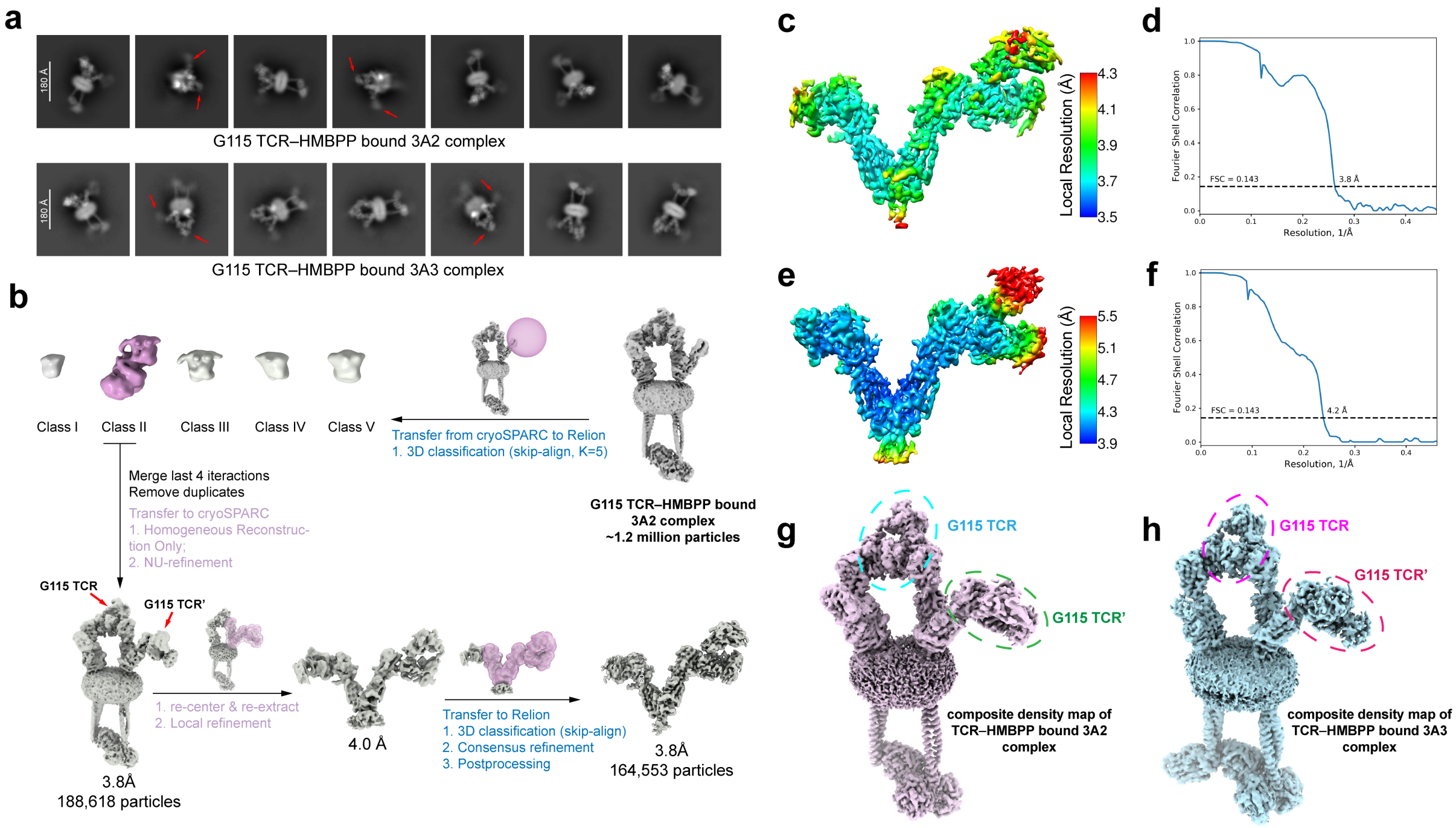

Supplementary Fig. S12 Cryo-EM data processing and analysis of the two Vγ9Vδ2 TCRs engaging the ectodomain of the pAg-bound BTN complexes. a, Selective 2D class averages indicate two TCRs bind ectodomains. Upper panel: the G115 TCR–HMBPP bound 3A2 complex; lower panel: the G115 TCR–HMBPP bound 3A3 complex. **b,** Data processing workflows for the G115 TCR–HMBPP bound 3A2 complex. A similar workflow was applied to the dataset of the G115 TCR–HMBPP bound 3A3 complex. **c,d,** Local resolution estimation (**c**) and Fourier shell correlation (FSC) curves (**d**) for the G115 TCR–HMBPP bound 3A2 complex. The map is contoured at 9 σ. **e,f,** Local resolution estimation (**e**) and Fourier shell correlation (FSC) curves (**f**) for the G115 TCR–HMBPP bound 3A3 complex. The map is contoured at 7 σ. **g,h,** Composite density maps of the G115 TCR–HMBPP bound 3A2 complex contoured at about 7 σ (**g**) and G115 TCR–HMBPP bound 3A3 complex contoured at about 7 σ (**h**). The additional locally refined maps used for combination are shown in **Supplementary Fig. S9**.

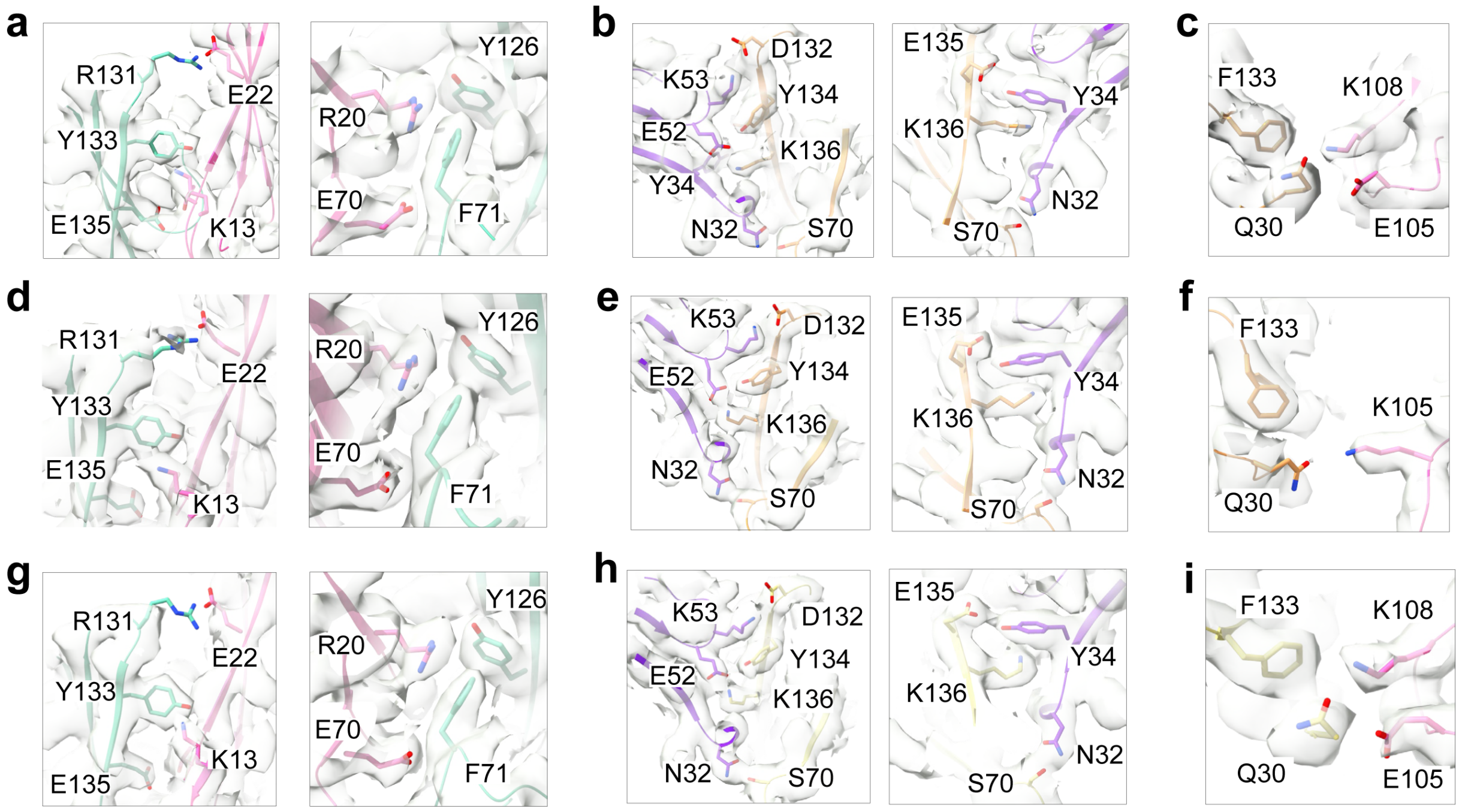

Supplementary Fig. S13 Representative cryo-EM maps of pAg-bound Vγ9Vδ2 TCR–BTN complexes. a-c, The interface between G115 TCR and the 3A2 complex: Close-up views of the interface between IgV_{BTN2A1} and the Vγ9 domain (**a**), IgV_{BTN3A2} and the Vδ2 domain (**b**), and IgV_{BTN3A2} and the Vγ9 domain (**c**). **d–f,** The interface between MOP TCR and the 3A2 complex: Close-up views of the interface between IgV_{BTN2A1} and the Vγ9 domain (**d**), IgV_{BTN3A2} and the Vδ2 domain (**e**), and IgV_{BTN3A2} and the Vγ9 domain (**f**). **g–i,** The interface between G115 TCR and the 3A3 complex. Close-up views of the interface between IgV_{BTN2A1} and the Vγ9 domain (**g**), IgV_{BTN3A3} and the Vδ2 domain (**h**), and IgV_{BTN3A3} and the Vγ9 domain (**i**). The map is contoured at 9 σ (**a–c**), 6 σ (**d–f**), and 9 σ (**g–i**), respectively. Residue K105 in TCR-MOP (**f**) structurally corresponds to K108 in G115 TCR (c and i), reflecting differences in the Vγ9 leader sequence and CDR3γ length.

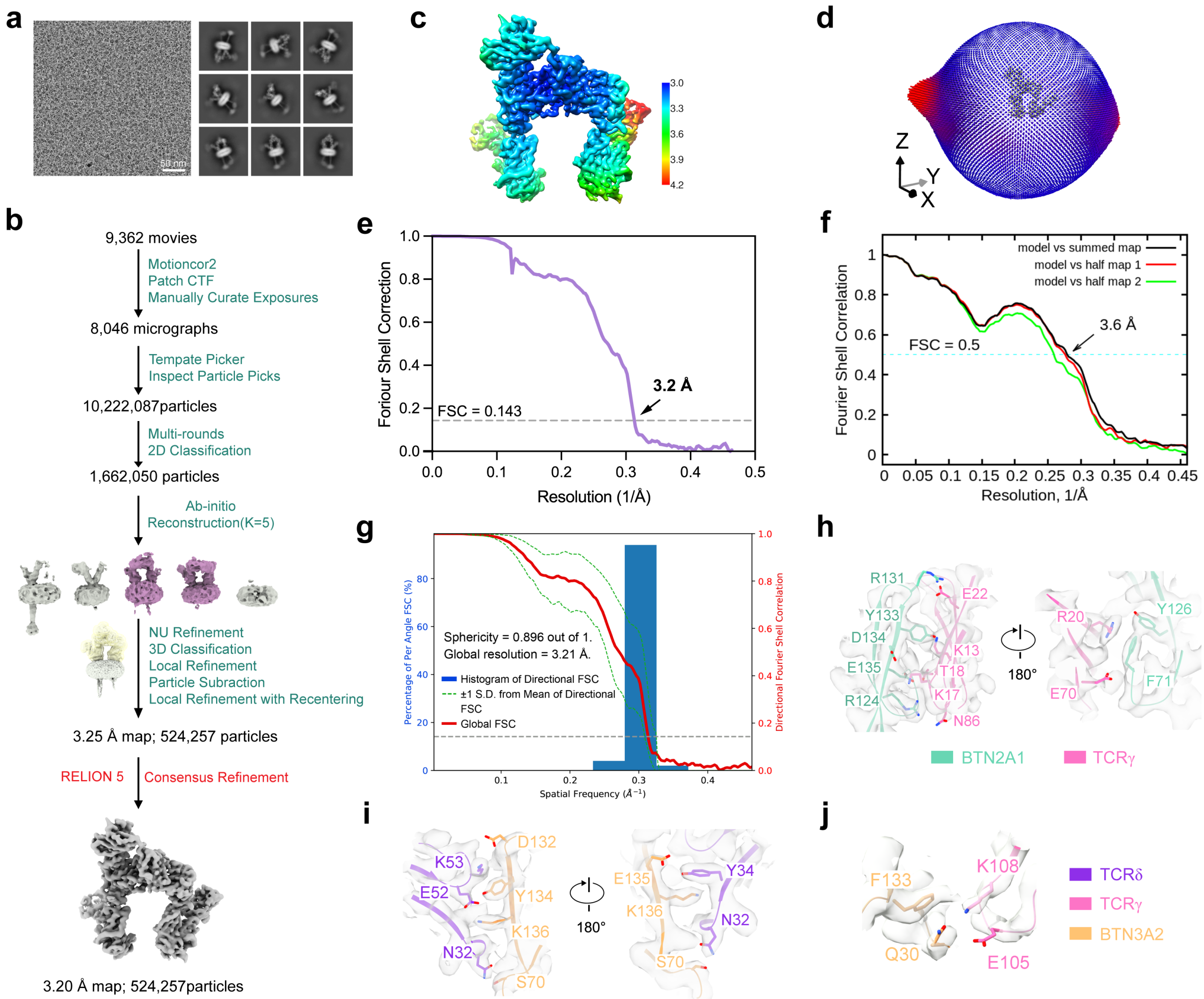

Supplementary Fig. S14 Cryo-EM data processing and analysis of the G115 TCR–AAR complex. a–b, Representative cryo-EM images, 2D class averages, and data processing workflows for the G115 TCR–AAR complex. AAR complex, a 3A2 complex variant with mutations H381R in BTN3A1 and R477A/T510A in BTN2A1 B30.2 domains to disrupt pAg binding. **c–d,** Local resolution estimation (**c**) and angular distribution (**d**) for the final reconstructions. The map is contoured at 9 σ in (**c**). **e,** Fourier shell correlation (FSC) curves for the final refinement. The dashed line indicates FSC = 0.143. **f,** Model versus map FSC curves for the G115 TCR–AAR complex. **g,** 3DFSC plots for the G115 TCR–AAR complex. **h–j,** Close-up views of the interfaces between IgV_{BTN2A1} and the Vγ9 domain (**h**), IgV_{BTN3A2} and the Vδ2 domain (**i**), and IgV_{BTN3A2} and the Vγ9 domain (**j**) for the G115 TCR–AAR complex. The map is contoured at 9 σ.

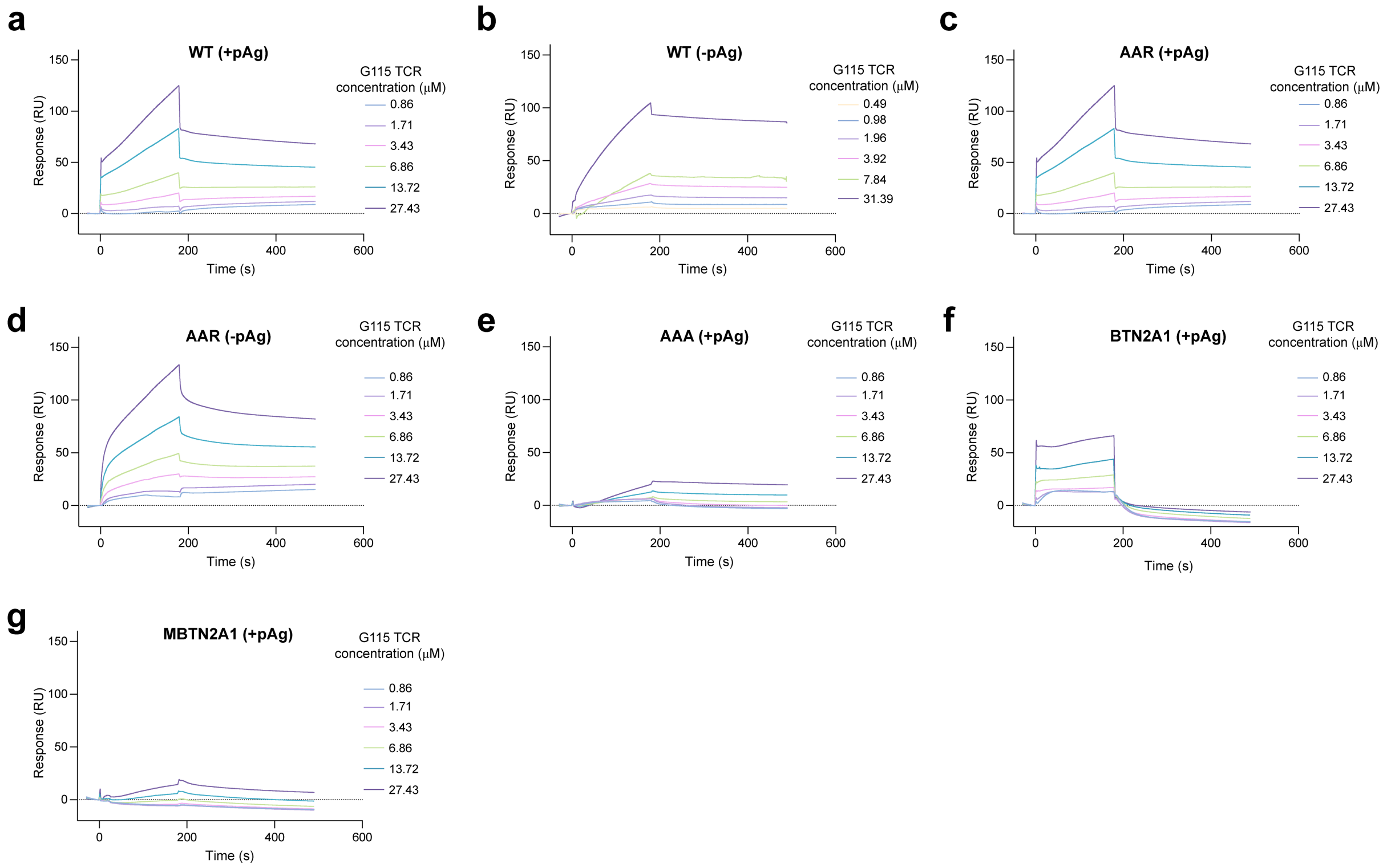

Supplementary Fig. S15 Surface plasmon resonance (SPR) analysis of binding affinities of G115 TCR towards different BTN molecules. a–g, Sensorgrams of SPR showing the binding of soluble G115 TCR ectodomain to immobilized different BTN molecules. The presence or absence of HMBPP is indicated as +pAg or -pAg, respectively. WT: wild-type. AAR complex, a 3A2 complex variant with mutations H381R in BTN3A1 and R477A/T510A in BTN2A1 B30.2 domains, designed to disrupt HMBPP binding. AAA complex, a 3A2 complex variant carrying the R124A/Y133A mutations in BTN2A1 and D132A in BTN3A2. MBTN2A1, a BTN2A1 variant carrying E63A/R65A/F71A/K79A/R84A/E87A/R131A/E135A mutations in BTN2A1. RU: resonance unit.

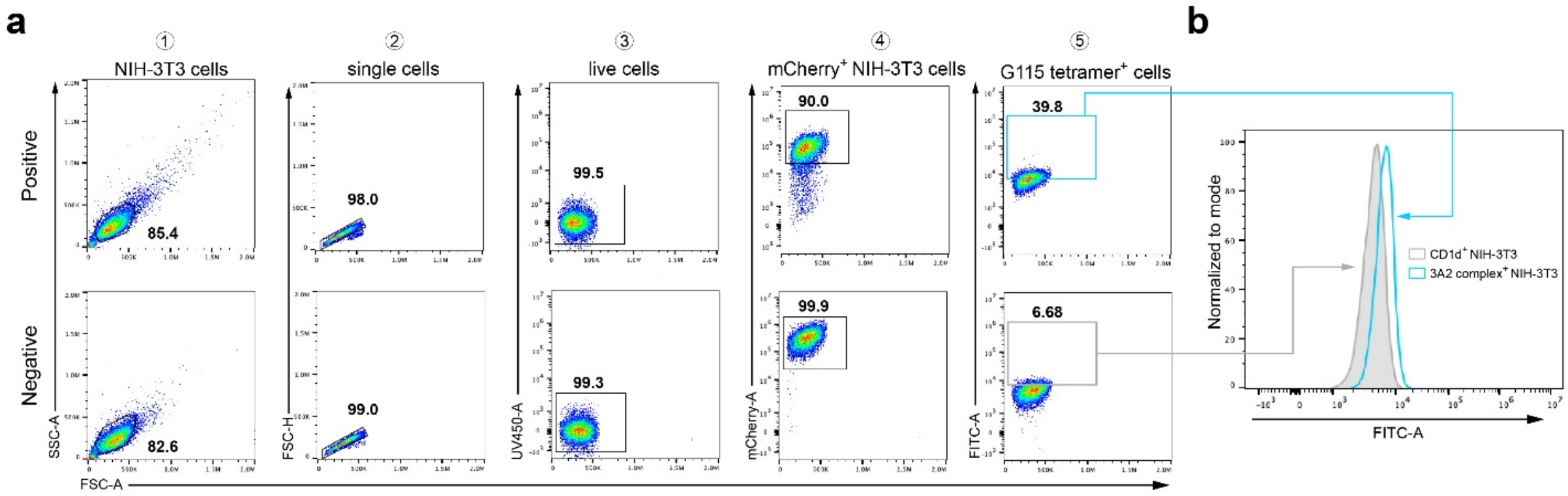

Supplementary Fig. S16 Gating strategy for analyzing tetramer-stained cells. a, CD1d-expressing NIH-3T3 cells were used as a negative control to establish the gating threshold for tetramer-positive events in NIH-3T3 cells expressing the 3A2 complex. **b,** Histograms of tetramer staining shows positive cells expressing 3A2 complex and negative cells expressing CD1d.

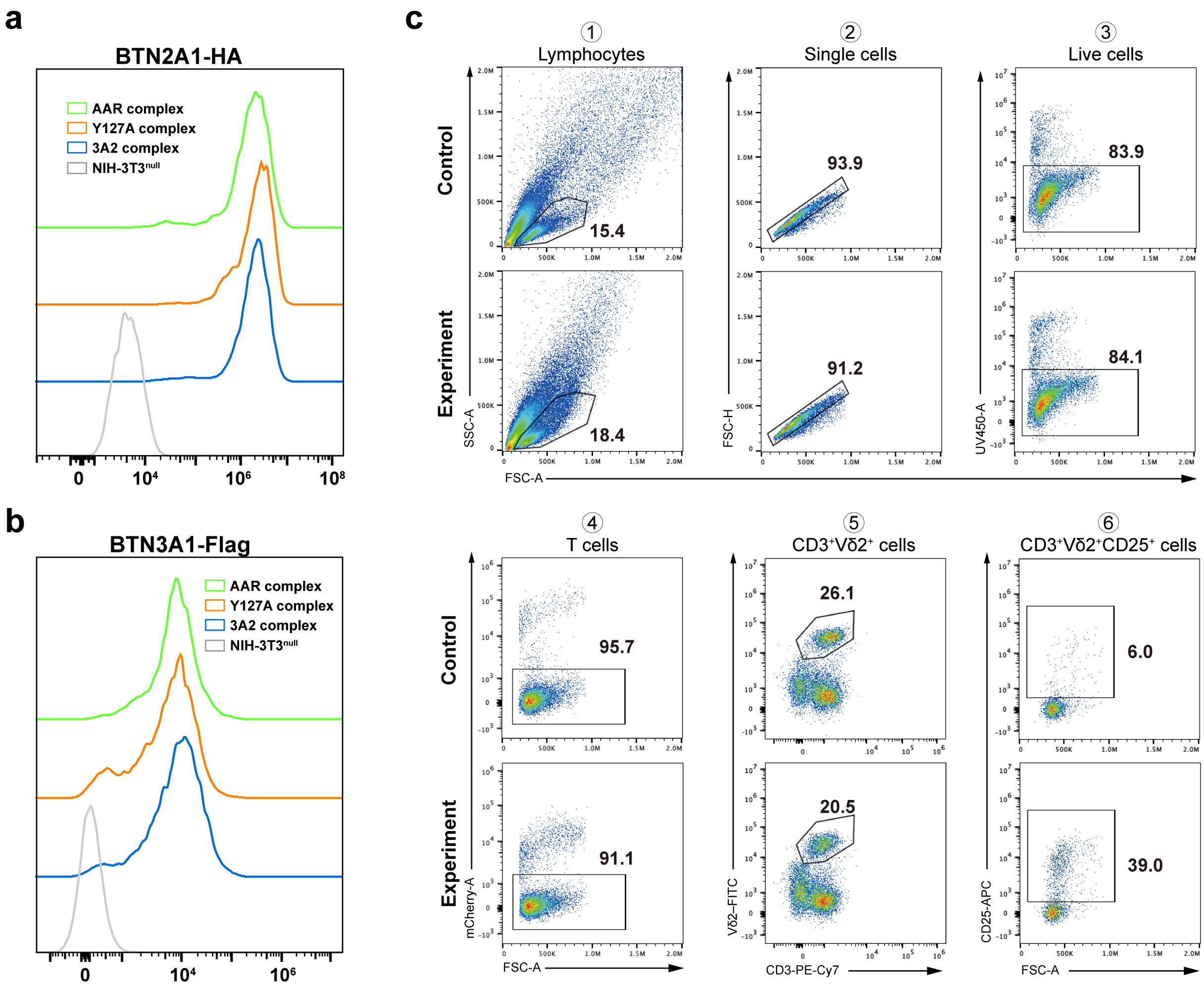

Supplementary Fig. S17 Surface expression of BTN2A1 and BTN3A in transduced NIH-3T3 cells and gating strategy for identifying CD25⁺ Vγ9Vδ2 T cells in functional assays. **a,** Anti-BTN2A1-HA staining of each cell line with untransduced NIH-3T3 cells serving as negative controls (gray). **b,** anti-BTN3A1-Flag staining of each cell line with untransduced NIH-3T3 cells serving as negative controls (gray). **c,** Gating strategy for identifying CD25$^+$ Vγ9Vδ2 T cells. The gating threshold for CD25$^+$ events in Vγ9Vδ2 T cells was established using Vγ9Vδ2 T cells co-cultured with BTN-expressing NIH-3T3 cells or K562 cells as the experimental condition. Vγ9Vδ2 T cells co-cultured with CD1d-expressing NIH-3T3 cells served as the negative control in Fig. 4e.

Supplementary Table S1 Cryo-EM data collection, refinement and validation statistics.

	3A2 complex with HMBPP	G115 TCR–3A2 complex with HMBPP	MOP TCR–3A2 complex with HMBPP	3A3 complex with HMBPP	G115 TCR–3A3 complex with HMBPP	G115 TCR–AAR complex	3A2 complex oligomer
	PDB 8ZHR (Overall)	PDB 8ZK0 (Overall)	PDB 8ZJX (Overall)	PDB 8ZJZ (Overall)	PDB 8ZJY (Overall)	PDB 9IRD (ECD)	PDB 9IY6 (ECD)
	EMD-60114 (Overall)	EMD-60162 (Overall)	EMD-60159 (Overall)	EMD-60161 (Overall)	EMD-60160 (Overall)	EMD-60811 (ECD)	EMD-60986 (ECD)
	EMD-60164 (ECD focused)	EMD-60170 (ECD focused)	EMD-60178 (ECD focused)	EMD-60167 (ECD focused)	EMD-60173 (ECD focused)		
	EMD-60166 (ICD focused)	EMD-60171 (ICD focused)	EMD-60177 (ICD focused)	EMD-60168 (ICD focused)	EMD-60174 (ICD focused)		
		EMD-60172 (TCR-BTN2A1 focused)		EMD-60169 (B30.2 focused)	EMD-60175 (B30.2 focused)		
Data collection and Processing							
Microscopy	Krios (Thermo Scientific)						
Voltage (kV)	300						
Camera	Falcon 4i	K3					
Magnification	165,000	81,000					
Pixel size at detector (Å/pixel)	0.72	1.087	1.0773	1.087	1.087	1.0773	1.0773
Total electron exposure (e-/Å²)	50						
Number of frames collected during exposure	N/A	32					
Defocus range (μm)	-1.0 to -2.0						
Automation software	EPU						
Energy filter slit width (eV)	10	20					
Micrographs collected (no.)	20,263	18,233	11,974	9,466	9,896	9,362	10,822
Micrographs used (no.)	16,185	14,293	10,958	8,527	8,784	8,046	9,246
Reconstruction							
Initial particle images (no.)	1,538,617	2,108,010	1,444,926	1,401,812	1,023,733	1,662,050	3,123,658
Final particle images (no.)	556,413	888,747	572,509	426,857	413,213	534,257	229,039
Point-group	C1	C1	C1	C1	C1	C1	C1
Resolution (Å)	2.96 (Global)	3.40 (Global)	3.40 (Global)	3.81(Global)	3.71 (Global)		
	2.90 (ECD)	3.20 (ECD)	3.10 (ECD)	3.56 (ECD)	3.27 (ECD)	3.20 (ECD)	11.36 (ECD)
	2.73 (ICD)	3.00 (ICD)	3.17 (ICD)	3.48 (ICD)	3.27 (ICD)		
				3.30 (B30.2)	3.40 (B30.2)		
FSC threshold	0.143	0.143	0.143	0.143	0.143	0.143	0.143
Map sharpening B-factor (Å²)							
	-60 (ECD)	-80 (ECD)	-70 (ECD)	-120 (ECD)	-80 (ECD)	-60 (ECD)	
	-35 (ICD)	-45 (ICD)	-60 (ICD)	-80 (ICD)	-80 (ICD)		
				-80 (B30.2)	-65 (B30.2)		
Map sharpening method	B-factor sharpen	B-factor sharpen	B-factor sharpen	B-factor sharpen	B-factor sharpen	B-factor sharpen	
Model refinement							
Initial model used (PDB code)	8JYE, AF2 predicted structures	8DFW, 8ZHR	8ZHR	8ZHR, AF2 predicted structures	8DFW, 8ZJZ	8ZK0	8ZHR
Model composition							
Non-hydrogen atoms	14,017	17,445	17,407	15,542	15,542	10,010	
Protein residues	1,767	2,201	2,197	1,952	1,952	1,288	
Ligands	1	1	1	1	1	N/A	
B factors (Å²)							
Protein	69.27	77.6	62.69	21.01	50.1	67.92	
Ligand	29.05	42.36	71.32	1.23	15.32	N/A	
R.m.s deviations							
Bonds length (Å)	0.004	0.0027	0.0034	0.0036	0.0047	0.0031	
Bonds Angle (°)	0.61	0.58	0.59	0.56	0.66	0.62	
Refinement package	Phenix 1.20	Phenix 1.20	Phenix 1.20	Phenix 1.20	Phenix 1.20	Phenix 1.20	
- real or reciprocal space	Real space	Real space	Real space	Real space	Real space	Real space	
- resolution cutoff	0.5	0.5	0.5	0.5	0.5	0.5	
Model-Map scores							
- CC_volume/mask	0.83/0.82	0.82/0.81	0.75/0.76	0.67/0.64	0.78/0.80	0.75/0.75	
Validation							
MolProbity score	1.75	1.63	1.81	1.77	1.93	1.92	
CaBLAM outliers (%)	1.1	2.5	3.7	3.2	3.1	3.8	
Clashscore	8.67	6.83	8.14	7.63	8.95	10.49	
Poor rotamers (%)	0.2	0.63	0.37	0.77	0.53	0.09	
C-beta deviations (%)	0	0	0	0	0	0	
EMRinger score	2.53	2.1	2	2.58	1.85	2.35	
Ramachandran plot							
Preferred (%)	96.36	96.25	94.69	94.9	92.92	94.44	
Allowed (%)	3.53	3.7	5.26	5.1	7	5.52	
Outlier (%)	0.11	0.05	0.05	0	0.08	0.08	

Supplementary Video S2 pAg binding enhances the rigidity of the intracellular regions of the BTN molecules and drives dissociation of higher-order BTN oligomers into tetramers.
